# Synthesis and toxicity assessment of Fe_3_O_4_ NPs grafted by ∼ NH_2_-Schiff base as anticancer drug: modeling and proposed molecular mechanism through docking and molecular dynamic simulation

**DOI:** 10.1080/10717544.2020.1801890

**Published:** 2020-08-10

**Authors:** Rahime Eshaghi Malekshah, Bahareh Fahimirad, Mohammadreza Aallaei, Ali Khaleghian

**Affiliations:** a Department of Chemistry, College of Science, Semnan University, Semnan, Iran; b Department of Chemistry, Faculty of Science, Imam Hossein University, Tehran, Iran; c Biochemistry Department, Faculty of Medicine, Semnan University of Medical Sciences, Semnan, Iran

**Keywords:** Synthesis, nanocarrier, MTT assay, computational methods, molecular docking

## Abstract

Superparamagnetic iron oxide nanoparticles have been synthesized using chain length of (3-aminopropyl) triethoxysilane for cancer therapy. First, we have developed a layer by layer functionalized with grafting 2,4‐toluene diisocyanate as a bi‐functional covalent linker onto a nano-Fe_3_O_4_ support. Then, they were characterized by Fourier transform infrared, X-ray powder diffraction, field emission scanning electron microscopy, energy-dispersive X-ray spectroscopy, and VSM techniques. Finally, all nanoparticles with positive or negative surface charges were tested against K562 (myelogenous leukemia cancer) cell lines to demonstrate their therapeutic efficacy by MTT assay test. We found that the higher toxicity of Fe_3_O_4_@SiO_2_@APTS ∼ Schiff base-Cu(II) (IC_50_: 1000 μg/mL) is due to their stronger *in situ* degradation, with larger intracellular release of iron ions, as compared to surface passivated NPs. For first time, the molecular dynamic simulations of all compounds were carried out afterwards optimizing using MM+, Semi-empirical (AM1) and Ab-initio (STO-3G), Forcite Gemo Opt, Forcite Dynamics, Forcite Energy and CASTEP in Materials studio 2017. The energy (eV), space group, lattice parameters (Å), unit cell parameters (Å), and electron density of the predicted structures were taken from the CASTEP module of Materials Studio. The docking methods were used to predict the DNA binding affinity, ribonucleotide reductase, and topoisomerase II.

## 1. Introduction

Cancer is certainly one of the deadliest diseases in our society and serves as a primary targets in the field of medicinal chemistry. Many different kinds of compounds have been found to be active in restraining the reproduction of cancer cells, and some of them have been used in clinical treatments. Platinating agents, including cisplatin (CDDP), carboplatin (CBDCA), and oxaliplatin (L-OHP) have been widely used in the treatment of a wide spectrum of solid tumors (Abu-Surrah & Kettunen, 2006; Rabik & Dolan, 2007; Hayashi et al., 2016). To reduce side effects of chemotherapy in normal tissues, or decrease in the concentration of drug, the limited spectrum of activity and resistance caused by platinum compounds (Karasawa & Steyger, 2015), on the development of design and synthesis of new non-platinum compounds with potential cytotoxicity have focused in recent years (Yilmaz et al., 2016). In recent decades, inorganic magnetic core–shell nanoparticles (MNPs) as the fascinating area of green chemistry have received considerable attention using environmentally safe reagents and clean synthetic procedures (Dehghani et al., 2013; Esmaeilpour et al., 2014). Studies on various nanomaterial have shown that magnetic nanoparticles (MNPs) as an eco-friendly metal oxide have great potential in modern medical applications, including controlled drug and gene delivery systems and improve medical effect of cancer therapy via magnetic hyperthermia, and radiotherapy methodologies (Mancarella et al., 2015; Nigam & Bahadur, 2016; Ranmadugala et al., 2017). Photodynamic therapy (PDT), one of the use of MNPs is mainly taken for cancer diagnosis, selective therapy functions, and anticancer therapy (Nam et al., 2016; Choi et al., 2018). Photodynamic therapy, a major challenge in nanotechnology and nanomedicine, is with minimal side effects (Huang et al., 2011; Yin et al., 2012). Due to properties of the water-soluble photosensitizer MB, Fe_3_O_4_@mSiO_2_(MB)-FA MNPs were synthesized. After treated cells containing different concentrations, the tumor site was exposed to visible light at 650 nm laser. The results demonstrated that system could effectively be in NIR fluorescence imaging (Zhao et al., 2014). Another researches, the MNPs with Chlorin e6 were designed to fluorescence imaging and PDT to diagnose cancer and treat cancer (Huang et al., 2011; Li et al., 2018).

**Scheme 1.**
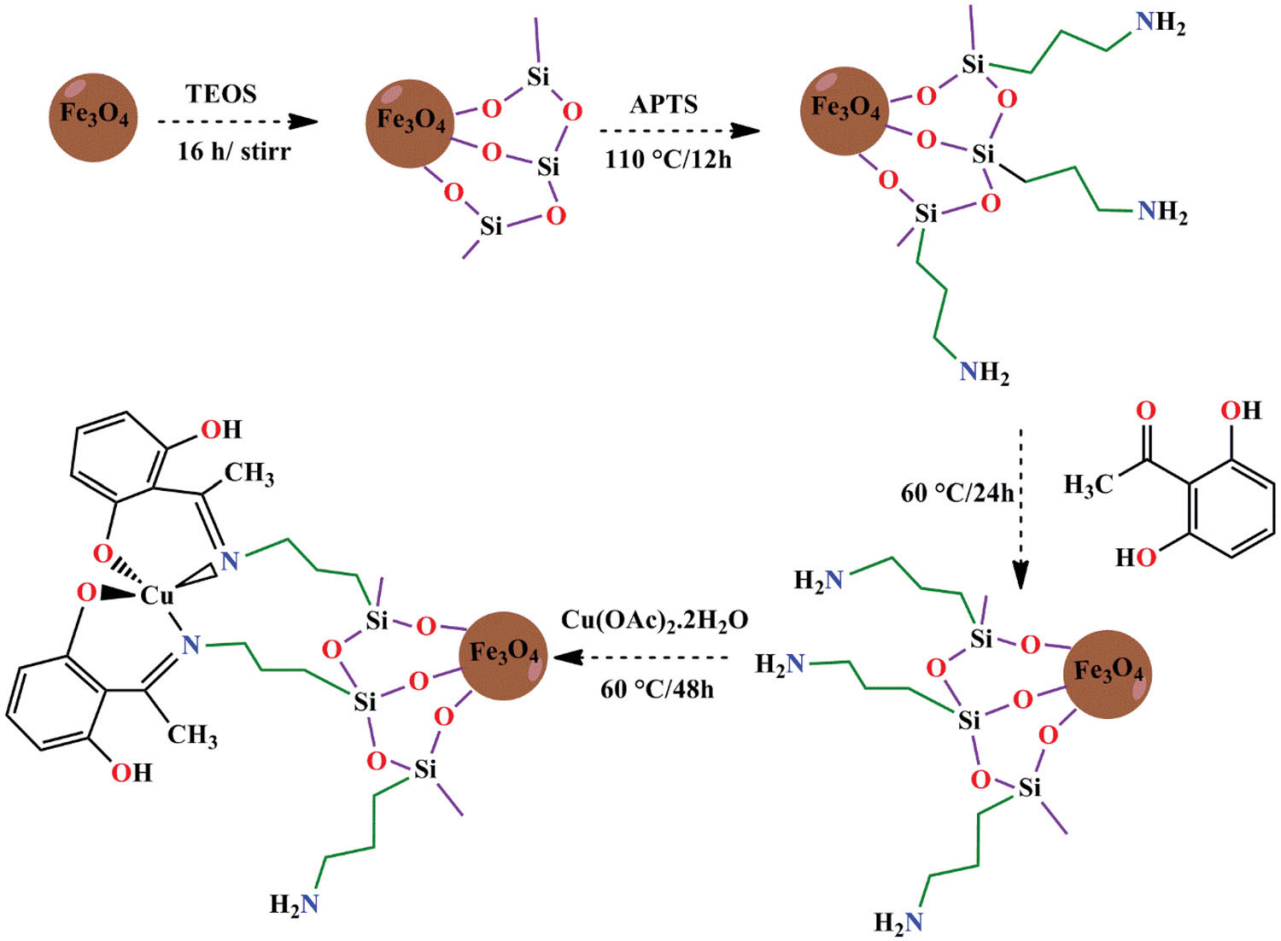
The synthesize of Fe_3_O_4_@SiO_2_@APTS NPs functionalized by ∼ NH_2_-Schiff and its Cu(II).

Niemirowicz et al. reported the synthesis of core–shell magnetic nanostructures with terminal propylo-amine groups cathelicidin and LL-37 peptide. Then, anticancer activity of MNPs is functionalized by cathelicidin LL-37 against colon cancer culture (DLD-1 cells and HT-29 cells). Results suggest that LL-37 peptide linked to MNPs (MNP@LL-37) have a therapeutic role with a higher rate in treatment and management of cancer compared with treatment using free LL-37 peptide (Niemirowicz et al., 2015). Schiff bases and their metal-based drugs have been great important candidates in coordination chemistry due to their structural similarities with natural biological compounds. They can be synthesized with different metal ions that show desirable activities via azomethine nitrogen atom (–C=N–) (Irfan et al., 2020; Revathi et al., 2020). Creation of new Schiff base ligands, as monodentate or multidentate chelating ligands, displays a significant role in antimicrobial activity, antioxidant activity, and cytotoxic effects (Jiang et al., 2020; Tavassoli et al., 2020). Numerous methods have been reported for the preparation of Schiff base (Chen et al., 2014). Fe_3_O_4_/SiO_2_/APTS (∼NH_2_) was synthesized and then functionalized by Schiff base complex Cu(II). The results of apoptosis study of Schiff base and complex nanoparticles showed apoptosis percentage of the nanoparticles increased upon increasing the thickness of Fe_3_O_4_ shell on the magnetite core (Malekshah et al., 2020). In this research, we decided to prepare and characterize Schiff base onto the surface of a novel magnetic nanosystem (iron oxide core) with (3-aminopropyl)triethoxysilane layer (a bridge between the surface of Fe_3_O_4_ nanoparticles and complex-Schiff base) as a novel method, green and recyclable heterogeneous catalyst (Scheme 1). The molecular dynamic simulations and molecular docking studies of all compounds were performed. In addition, the cytotoxicity properties of compounds were also investigated.

## 2. Experimental

### 2.1. Chemicals and instruments

All materials were purchased from Sigma-Aldrich (St. Louis, MO). FT-IR, XRD, SEM, and VSM were recorded on a SHIMADZU UV-1650PC (Kyoto, Japan), a Bruker D8000 (Bremen, Germany) in a scanning range of 2*θ* = 10–90° and CuK_α_ radiation, HITACHI S-4160 (Chiyoda City, Japan), EDX of the samples was determined using a Philips XL-30 energy-dispersive X-ray spectroscope (Amsterdam, Netherlands) and a vibrating sample magnetometer (VSM) MDKFD, respectively. Cell lines were obtained from National Cell Bank of Iran (NCBI)-Pasteur Institute of Iran (Tehran, Iran). Dulbecco’s modified eagle medium-high glucose (DMEM), fetal bovine serum (FBS) and penicillin–streptomycin were obtained from Gibco BRL (Life Technologies, Paisley, Scotland). The culture plates were obtained from Nunc (Roskilde, Denmark). MTT was purchased from Sigma. Chem. Co. (Munich, Germany).

#### 2.1.1. Preparation of the magnetic Fe_3_O_4_ and Fe_3_O_4_@SiO_2_ nanoparticles

Six grams of FeCl_3_·6H_2_O and 2 g FeCl_2_·4H_2_O in 100 mL deionized water were used. After sonicating 20 min, 10 mL of ammoniac solution under a nitrogen atmosphere and temperature at 90 °C was added dropwise into a mixture solution. After stirring about 1 h, the MNP black precipitate was separated by external magnet. Finally, Fe_3_O_4_ nanoparticles was washed with the double distilled water and dried at 60 °C overnight. One gram of Fe_3_O_4_ was dispersed in 100 mL of the ethanol/H_2_O with ultrasonication. Subsequently, 1.5 mL of ammoniac solution was added and dispersed with ultrasonication for 30 min. In the next step, 10.5 mL TEOS was added into the mixture and stirred for 16 h. The suspension is filtrated and washed with ethanol and deionized water for three times and dried in oven at 60 °C overnight.

#### 2.1.2. Preparation of Fe_3_O_4_@SiO_2_@APTS core shell

One gram of Fe_3_O_4_@SiO_2_ was added in 25 mL toluene and dispersed by ultrasonic. Then, (3-aminopropyl) triethoxysilane (2 mL) was added into the mixture and the solution was refluxed at 110 °C for 12 h. The resulting suspension was collected and washed with ethanol and deionized water for three times and finally dried in a vacuum at 50 °C overnight (Fahimirad et al., 2019).

#### 2.1.3. Preparation of Fe_3_O_4_@SiO_2_@APTS ∼ NH_2_-Schiff base nanoparticles

For preparation of Fe_3_O_4_@SiO_2_@APTS ∼ NH_2_**-**Schiff base nanoparticles, Fe_3_O_4_@SiO_2_∼NH_2_ (0.4 g) was dissolved in 20 mL of methanol. Then, 2,4-dihydroxybenzaldehyde (0.22 g) in methanol (10 mL) added to the solution of Fe_3_O_4_@SiO_2_@APTS ∼ NH_2_ and the resultant mixture was under refluxed at 60 °C for 24 h. The nanoparticles were washed with methanol to remove no reacted species and dried (Roozbahani et al., 2019).

#### 2.1.4. Synthesis of Fe_3_O_4_@SiO_2_@APTS ∼ NH_2_ Schiff base@Cu(II) nanoparticles

Solution of Cu(OAc)_2_·2H_2_O (0.2 g) in methanol was added to Fe_3_O_4_@SiO_2_@APTS-Schiff base (0.2 g, 20 mL MeOH), then the mixture was refluxed at 60 °C for 48 h. The resulting product was separated by filtration and washed with acetone and water (10 mL) and deionized water, then dried in vacuum at 80 °C overnight.

### 2.2. Biological studies

#### 2.2.1. Preparation of cell culture

The cell lines K562 (a human erythroleukemia cancer) were cultured in DMEM with 10% heat-inactivated FBS (Gibco, Invitrogen, Carlsbad, CA) 104 U/mL penicillin–streptomycin as antibiotics with humidified air containing of 5% CO_2_ atmosphere at 37 °C (Heraeus, Hanau, Germany). The cells should have 80–90% confluence before they are harvested and plated for the experiments. The cell lines K562 (a human erythroleukemia cancer) were cultured in minimum essential medium of RPMI 1640 medium with inactivated 10% FBS (Sigma, Munich, Germany), 104 U/mL penicillin–streptomycin as antibiotics (Biosera, Ringmer, UK) in plates and incubated in incubator at 37 °C with 5% CO_2_ (Heraeus, Hanau, Germany) (Malekshah et al., 2019).

#### 2.2.2. Assessment of cytotoxicity using MTT assay

The cytotoxicity effect of all compounds was determined in K562 using MTT assay (Malekshah et al., 2018). The cells were seeded at a density of 1 × 10^3^ per well into 96 well tissue culture plates. The amounts of nanoparticles at six different concentrations of 1, 10, 25, 50, 100, and 1000 μg/mL were added to the wells after reaching the state of 80% confluence. The plates were incubated in a humidified atmosphere 5% CO_2_. After 48 h, 20 µL of MTT (5 mg/mL) was added to each well and further incubated for 4 h. The medium of the plate was removed and 100 μL of DMSO was added to dissolve the MTT formazan precipitate. The absorbance of samples was determined at 570 nm. The cytotoxicity effect of Fe_3_O_4_@SiO_2_@APTS∼, Fe_3_O_4_@SiO_2_@APTS ∼ Schiff base and Fe_3_O_4_@SiO_2_@APTS ∼ Schiff base-Cu(II) was determined by MTT method.

### 2.3. Computational methods

The Fe_3_O_4_ optimization was done using modules Dmol3 and CASTEP in Materials studio2017. The Fe_3_O_4_@SiO_2_@APTS∼, Fe_3_O_4_@SiO_2_@APTS ∼ Schiff base, and Fe_3_O_4_@SiO_2_@APTS ∼ Schiff base-Cu(II) were optimized using MM+, Semi-empirical (AM1), and Ab-initio (STO-3G). The modules Forcite Gemo Opt, Forcite Dynamics, Forcite Energy, and CASTEP in Materials studio2017 were used for the final calculation of core–shell linker. The energy (eV), space group, lattice parameters (Å), unit cell parameters (Å), and electron density of the predicted structures were taken from the CASTEP module of Materials Studio.

### 2.4. Molecular docking of the compounds with DNA duplex of sequence d (ACCGACGTCGGT)_2_ (PDB ID: 1BNA), ribonucleotide reductase (3hne), and topoisomerase II (PDB ID: 4fm9)

To understand antitumor activity and the binding site of the target-specific region of compounds, we used molecular docking simulation (Malekshah & Khaleghian, 2019). The 3D crystal structures of 1BNA with the sequence, topoisomerase II (PDB ID: 4fm9), anticancer drugs (doxorubicin, mitoxantrone, and trifluridine), ribonucleotide reductase (PDB ID: 3hne1), and triapine were retrieved from RCSB Protein Data Bank and Pubchem. Also, to make cellular membrane, VMD was used by selecting the Extensions → Modeling → Membrane Builder menu item in cellular membrane was built on the *x* and *y* axes and CHARMM topology. In the last, it is converted into pdb format.

The PDB format of synthesized compounds was taken from DMol3 and Castep in Materials studio2017. First of all, the hetero-atoms including water molecules around the duplex were merged using the AutoDock tools, then polar hydrogen atoms, Kollman united atom type charges and Gasteiger partial charges were assigned to the receptor molecule and saved in PDBQT file (Malekshah et al., 2019). All the docking simulations were defined by using a grid box with 74 × 64 × 117 Å points with a grid-point spacing of 0.375 Å for BNA, 126 × 126 × 126 Å with a grid-point spacing of 0.908 Å for Top II, 126 × 126 × 126 Å with a grid-point spacing of 0.980 Å for ribonucleotide reductase and 126 × 126 × 126 Å with a grid-point spacing of 0.602 Å for lipid. The molecular docking using a Lamarckian genetic algorithm method was engaged to study this interaction. The best optimized model having lowest energy was picked up from the one minimum energy (RMSD = 0.0) from the 100 runs. Then, the interactions and their binding modes with compounds were analyzed using an AutoDock program 1.5.6, UCSF Chimera1.5.3 software, Discovery Studio 2017R2 client from Accelrys and DSVisualizer2.0.

## 3. Results and discussion

### 3.1. Infrared spectra of compounds

**Figure 1.**
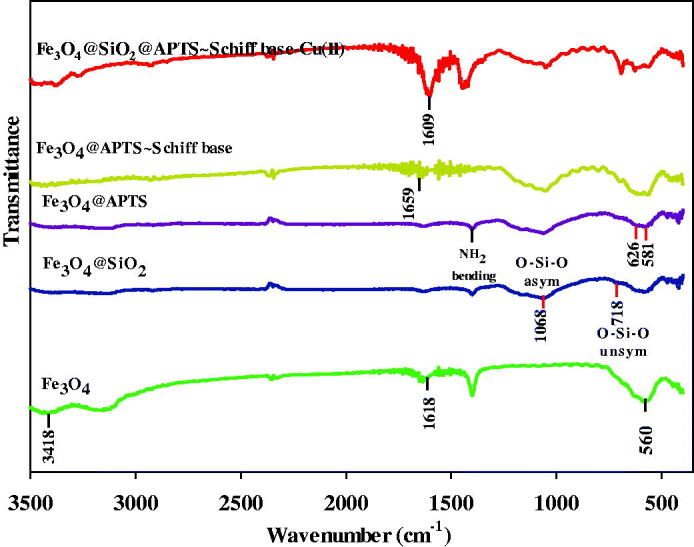
FTIR spectra of bare (a) Fe_3_O_4_ MNPs; (b) Fe_3_O_4_@SiO_2_; (c) Fe_3_O_4_ MNPs treated by APTES; (d) Fe_3_O_4_ MNPs coated by Schiff base; (e) Fe_3_O_4_@SiO_2_@APTS ∼ Schiff base-Cu(II).

The stretching vibrations in 3418 and 1618 cm^−1^ are attributed to the O–H stretching and deforming vibrations of adsorbed water (Figure 1(a)). The absorption around 560 cm^−1^ of Fe_3_O_4_ is indexed to Fe–O vibration (Fahimirad et al., 2018). In addition, the antisymmetric and symmetric stretching vibration of Si–O–Si and O–Si stretching vibrations are observed at 1068 and 784 cm^−1^, respectively (Figure 1(b)). Evidently, it indicates that the silica has been successfully coated on the surface of superparamagnetic Fe_3_O_4_ NPs (Figure 1(b)). Figure 1(c) shows the Fe–O (stretching vibration) at 626 and 581 cm^−1^, Si–O–Si (asymmetric stretching) in the region 1000–1180 cm^–1^ and C–N (stretching vibration) at 1401 and N–H (bending) at 1639 cm^−1^, respectively (Esmaeilpour et al., 2012). In the FT-IR spectrum of Fe_3_O_4_@SiO_2_/ligand observed at 1659 cm^−1^ is assigned to the C=N stretching frequency of the newly formed azomethine group and another new band appears around 1509 cm^−1^ which is allocated to the aromatic C=C stretch (Figure 1(d)). Also, O–H stretching band of Fe_3_O_4_@SiO_2_/ligand is observed at 3447 cm^−1^. In addition, the absorption peak at 567 cm^−1^ is assigned for Fe–O stretching vibration in Fe_3_O_4_ (Tavassoli et al., 2020). The *υ*(C=N) absorption of the Schiff base shift is toward the lower frequency in complex (1609 cm^−1^), suggesting the coordination of the nitrogen with the metal (Figure 1(e)). The absorption peak at 563 cm^−1^ belonged to the stretching vibration mode of Fe–O bonds in Fe_3_O_4_. The presence of vibration bands in 3380 (O–H stretching), 2870–3100 (CH stretching), 1480–1600 (C=C aromatic ring stretching), and 1491 (CH_2_ bending) demonstrates the existence of ligand complex of Cu(II) on Fe_3_O_4_@SiO_2_ nanoparticles in the spectrum (Figure 1(e)). Also, the antisymmetric and symmetric stretching vibrations of Si–O–Si bond in oxygen–silica tetrahedron are observed at 1055 and 814 cm^−1^, respectively (Kohler et al., 2005).

### 3.2. XRD spectra

The crystalline or amorphous structure of compounds was determined by XRD analysis. Therefore, the XRD analysis was taken from samples of Fe_3_O_4_@SiO_2_ and Fe_3_O_4_@SiO_2_@APTS ∼ Schiff base-Cu(II) (Supplementary Figure 1). In two Figure 1S.a and 1S.b, peaks in 2*θ* = 30.1, 34.7, 42.3, 56.2, 57.1, and 62.5° can be seen that are in agreement with Fe_3_O_4_ nanostructures JCPDS no. 19-0629^15^. Also, given that the silica and Schiff base complex structures are amorphous, it is expected to be observed broad peak at 2*θ* = 20–30°. Figure 1S.b clearly shows the broad peak at 2*θ* = 20–30° that can confirm an amorphous silica shell and organic components in the synthesized structure.

### 3.3. VSM

The magnetic properties of synthesized sample are analysed by VSM at room temperature. Consequently, in order to obtain the magnetic strength Fe_3_O_4_@SiO_2_ and Fe_3_O_4_@SiO_2_@APTS ∼ Schiff base-Cu(II), the analysis was performed and based on the results in Figure 3, the maximum saturation magnetization (Ms) values for Fe_3_O_4_ and Fe_3_O_4_@SiO_2_@APTS ∼ Schiff base-Cu(II) were obtained at 61.60 and 36.23, respectively. According to the results presented in the Supplementary Figure 2, after the surface modification of Fe_3_O_4_ by SiO_2_@APTS ∼ Schiff base-Cu(II), the maximum saturation magnetization decreased that it indicates the formation of SiO_2_@APTS ∼ Schiff base-Cu(II) crust on the Fe_3_O_4_ core. Finally, Fe_3_O_4_@SiO_2_@APTS ∼ Schiff base-Cu(II) can be separated easily by using an applied magnetic field.

**Figure 2.**
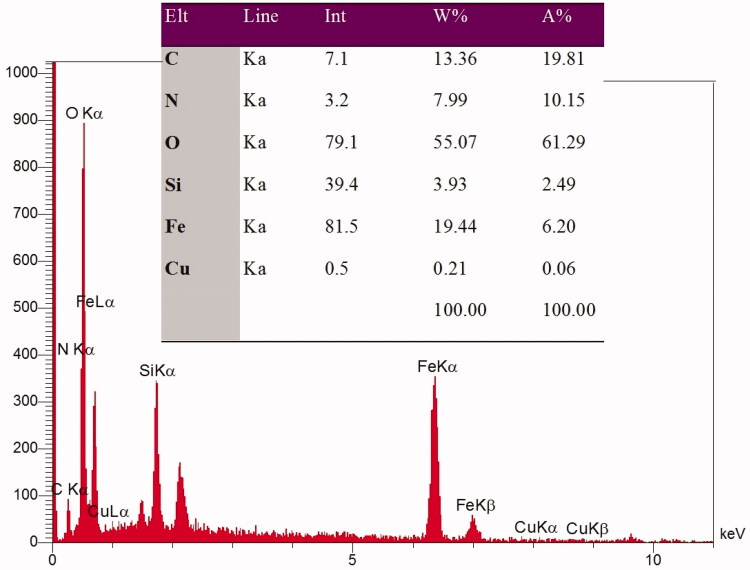
EDX spectra obtained for Fe_3_O_4_@SiO_2_@APTS ∼ Schiff base-Cu(II) nanoparticles.

### 3.4. SEM and EDX

The FE-SEM images could indicate the distribution of the particles and particle size in synthesizes samples. Therefore, FE-SEM was taken from Fe_3_O_4_@SiO_2_ and Fe_3_O_4_@SiO_2_@APTS ∼ Schiff base-Cu(II) nanoparticles. Supplementary Figure 3 shows a good distribution of the Fe_3_O_4_@SiO_2_ particles, indicating a lack of agglomeration in the synthesized Fe_3_O_4_@SiO_2_. Also, after modifying of the Fe_3_O_4_@SiO_2_ surface by APTS ∼ Schiff base-Cu(II) nanoparticles, it can be resulted that morphology of nanoparticles has not been changed and the particles are completely dispersed and uniformed. Also, the nanostructures in Supplementary Figures 3.1a and 3.1b have a particle size∼20 and about 25 nm, for Fe_3_O_4_@SiO_2_ and Fe_3_O_4_@SiO_2_@APTS ∼ Schiff base-Cu(II) nanoparticles, respectively. Also, the EDX spectra in Figure 2, conform element of Fe, C, N, Si, and Cu. These results confirm the accuracy of the synthesized sample.

**Figure 3.**
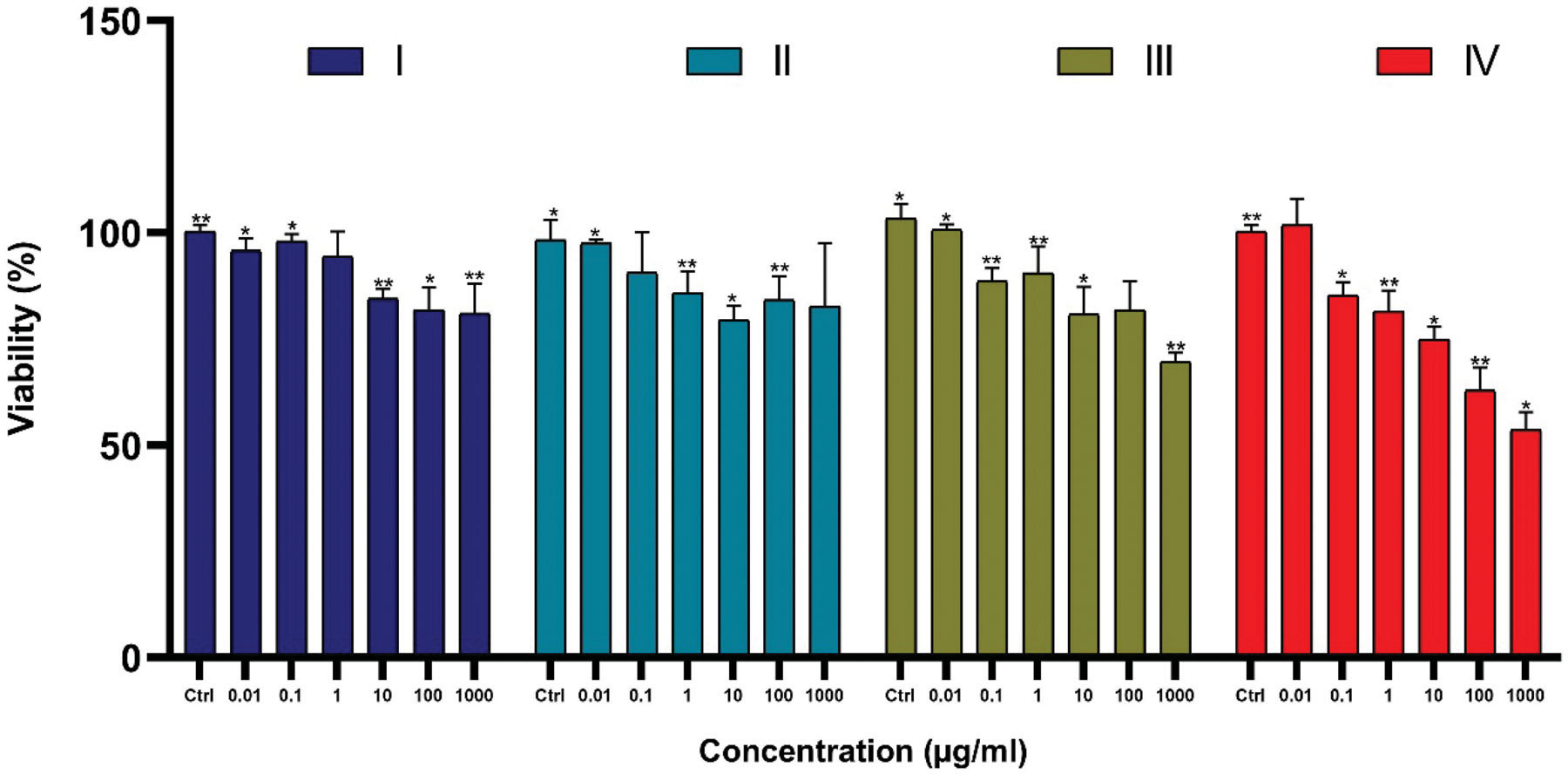
The anti-growth effect after the treatment with varying doses of Fe_3_O_4_@SiO_2_@APTS (A); Fe_3_O_4_@SiO_2_@APTS ∼ Schiff base (B); APTS ∼ Schiff base-Cu(II) (C) on K562 cell lines by the MTT. Data were normalized as a percentage of values of the control (*p<.05 and **p<.01).

### 3.5. TEM analysis

Supplementary Figure 4 shows the spherical morphology of the nanocomposite. These were taken from different parts of the sample (Supplementary Figure 4). According to the figure, the particles are well dispersed and the core–shell structure is clearly marked. In this figure, the core of the nanoparticles (Fe_3_O_4_) is dark regions and the shell structure (SiO_2_@APTS ∼ Schiff base-Cu(II)) is the lighter regions represent. Also, the figures clearly show the uniformity of the particle size of the nanoparticles. According to these images, the average particle size is approximately 25 nm, which is in agreement with SAM images.

## 4. Biological assay

### 4.1. Assessment of cytotoxicity using MTT assay

The cytotoxicity/viability of compounds was examined by conventional MTT assay in K562 cell lines and the results are shown in Figure 3. Our results indicated that the anticancer effects of the nanoparticles increased upon increasing the thickness of Fe_3_O_4_ shell on the magnetite core Fe_3_O_4_@ SiO_2_@ APTS ∼ Schiff base-Cu( II)>Fe_3_O_4_@SiO_2_@APTS ∼ Schiff base > Fe_3_O_4_@SiO_2_@APTS > Fe_3_O_4_@ SiO_2_. Also, the results show that toxicity of nanoparticles is dose dependent and prevent the cell growth and viability with increase in dose.

**Table 1.** CSP results showing list of unique crystal structures ranked on the basis of the calculated lattice energy and density.

Compound	Fe_3_O_4_	Fe_3_O_4_@SiO_2_	Fe_3_O_4_@SiO_2_@APTS	Fe_3_O_4_@SiO_2_@APTS ∼ Schiff base	Fe_3_O_4_@SiO_2_@APTS ∼ Schiff base-Cu(II)
Crystal system	Rhombohedral	Rhombohedral	Rhombohedral	Rhombohedral	Rhombohedral
Space group	*P*1	*P*1	*P*1	*P*1	*P*1
*a* (Å)	6.034	9.025	8.466	9.771	9.973
*b* (Å)	6.034	9.025	8.466	9.771	9.973
*c* (Å)	6.034	9.025	8.466	9.771	9.973
*α* (°)	60.00	90.000	90.000	90.000	90.000
*β* (°)	60.00	90.000	90.000	90.000	90.000
*γ* (°)	60.00	90.000	90.000	90.000	90.000
Cell volume	–	735.092	606.957	933.148	992.22
Final energy	–8688.084 eV	–11752.099	–12044.134 eV	–16508.423 eV	–18154.089 eV

## 5. Simulation methods

### 5.1. Crystal structure prediction (CSP)

We were carried out by NVT (constant particle number, constant volume, and constant temperature) and NPT (constant particle number, constant temperature, and constant pressure) 15 ns in the time and ensemble atomic simulation on compounds. Details about the data collection and figures of electron density of the optimized compounds are illustrated in Table 1 and Figure 4. The structures of all compounds crystallize in the *Rhombohedral* system with *P*1 space groups. The structure of Fe_3_O_4_@ SiO_2_@ APTS ∼  Schiff base-Cu(II) is tetrahedral with cell volume 992.22 Å^3^. Cell volume of Fe_3_O_4_@SiO_2_, Fe_3_O_4_@SiO_2_@ APTS, Fe_3_O_4_@ SiO_2_@ APTS ∼ Schiff base is 735.092, 606.957, and 933.148 Å^3^, respectively. This compound showed weak π–π stacking and hydrogens interactions between neighboring atoms (Supplementary Fig. 5), in addition, there is no π–π stacking and hydrogen interactions between neighboring atoms and cell data were estimated for Fe_3_O_4_@SiO_2_. The calculated frontier orbital density distributions of all nanoparticles are shown in Figure 4. The electrophilic and nucleophilic segments can be characterized by the levels of electron density observing in the different regions of our compound. The electron charge distributions of the HOMO and LUMO are plotted in Supplementary Figure 6. The HOMO and LUMO energies of Fe_3_O_4_@SiO_2_ are −20.00 and +1.462 eV, respectively. The HOMOs are largely localized on the Si of silica, while the LUMOs are localized on O of Si and Fe. The HOMO and LUMO energies of Fe_3_O_4_@SiO_2_@APTS are −19.83 and +5.808 eV, respectively. The HOMOs are largely localized on the OH of silica, while the LUMOs are localized on metal center. The HOMO and LUMO energies of Fe_3_O_4_@SiO_2_@APTS ∼ Schiff base are −22.893 and +5.877 eV, respectively. The HOMOs and LUMO are largely localized on the (OH of benzene ring and C of benzene ring) and (O of core), respectively. The HOMO and LUMO energies in Fe_3_O_4_@SiO_2_@APTS ∼ Schiff base-Cu(II) display one main band at −23.567 and +5.779 eV, respectively. The energy band gap, Δ*E*=*E*_LUMO_ – *E*_HOMO_ of Fe_3_O_4_@SiO_2,_ Fe_3_O_4_@SiO_2_@APTS, Fe_3_O_4_@SiO_2_@APTS ∼ Schiff base and Fe_3_O_4_@SiO_2_@APTS ∼ Schiff base-Cu(II) is 21.46, 25.63, 28.77, and 29.34 eV. Also, based on results of molecular dynamic simulations, the Fe_3_O_4_@SiO_2_@APTS ∼ Schiff base-Cu(II) has four-coordinated metal center with two nitrogen and two oxygen atoms from Schiff bases, resulting in a distorted tetrahedral coordination sphere. CASTEP results explain in Supplementary Figure 7. The values of band gap for Fe_3_O_4_@SiO_2_, Fe_3_O_4_@SiO_2_@APTS, Fe_3_O_4_@SiO_2_@APTS ∼ Schiff base, and Fe_3_O_4_@SiO_2_@APTS ∼ Schiff base-Cu(II) were 0.004, 0.027, 0.008, and 0.019 eV, respectively. The band gap of Fe_3_O_4_@SiO_2_@APTS and Fe_3_O_4_@SiO_2_@APTS ∼ Schiff base-Cu(II) is higher than Fe_3_O_4_@SiO_2_ and Fe_3_O_4_@SiO_2_@APTS ∼ Schiff base. The computational XRD and the experimental XRD are respectively shown in Supplementary Figs. 8 and 1. The optimized computational XRD is similar to that obtained with experimental XRD.

Figure 4.Perspective view and electron density of the optimized nano-particles.

**Figure:**
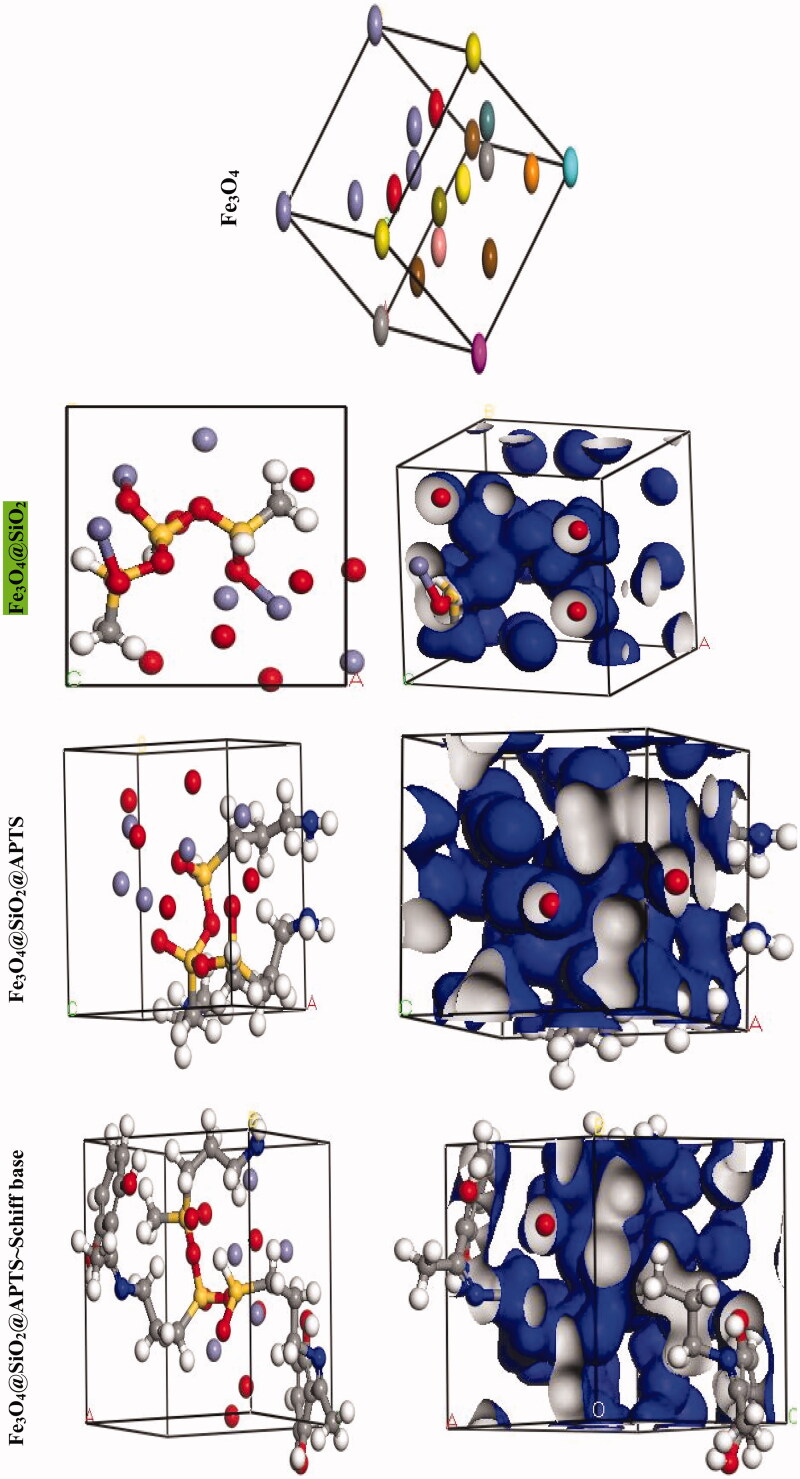

**Figure:**
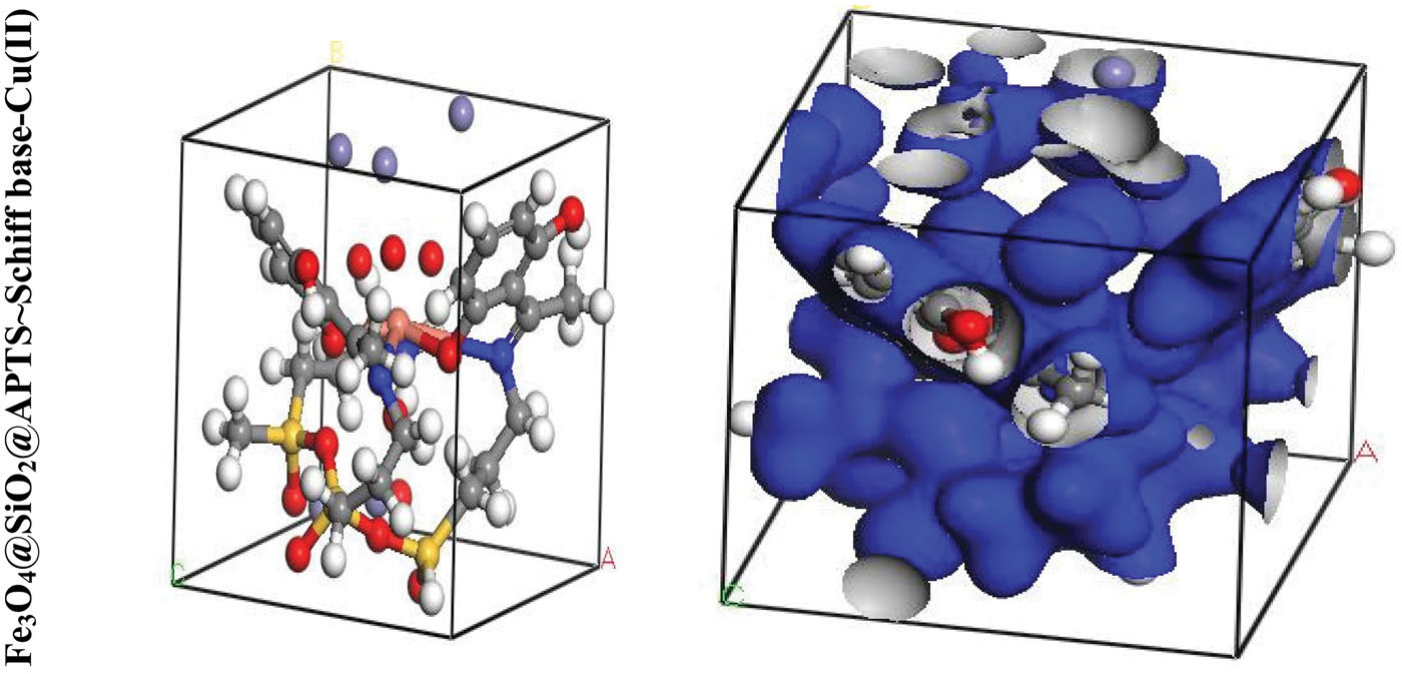

### 5.2. Molecular docking of the compounds with DNA, topoisomerase II, ribonucleotide reductase, and lipid

The molecular docking studies along with experimental studies could help to explore a compound as a potential drug candidate. The binding free energy values are dominated by the vdW + Hbond + desolv (kcal/mol) negative energy values, suggesting that the binding events of nanoparticles are a spontaneous process. The DNA-binding affinity of the Fe_3_O_4_@SiO_2_@APTS (–10.85 kcal mol^−1^) and mitoxantrone (–10.35 kcal mol^−1^) is stronger than the Fe_3_O_4_@SiO_2_ (–6.46 kcal mol^−1^), Fe_3_O_4_@SiO_2_@APTS ∼ Schiff base (–7.47 kcal mol^−1^), Fe_3_O_4_@SiO_2_@APTS ∼ Schiff base-Cu(II) (–7.59 kcal mol^−1^), and trifluridine (–5.56 kcal mol^−1^), respectively. The data and figures of compounds are shown in Figure 5 and Table 2. Based on a comparison of among results, all synthesized compounds showed significant affinity to DNA compared to trifluridine (as DNA–drug interaction). Also, compounds and anticancer drugs could bind to the minor groove of DNA.

Figure 5.Docking conformation of all synthesized compounds, mitoxantrone and trifluridine with BNA.

**Figure:**
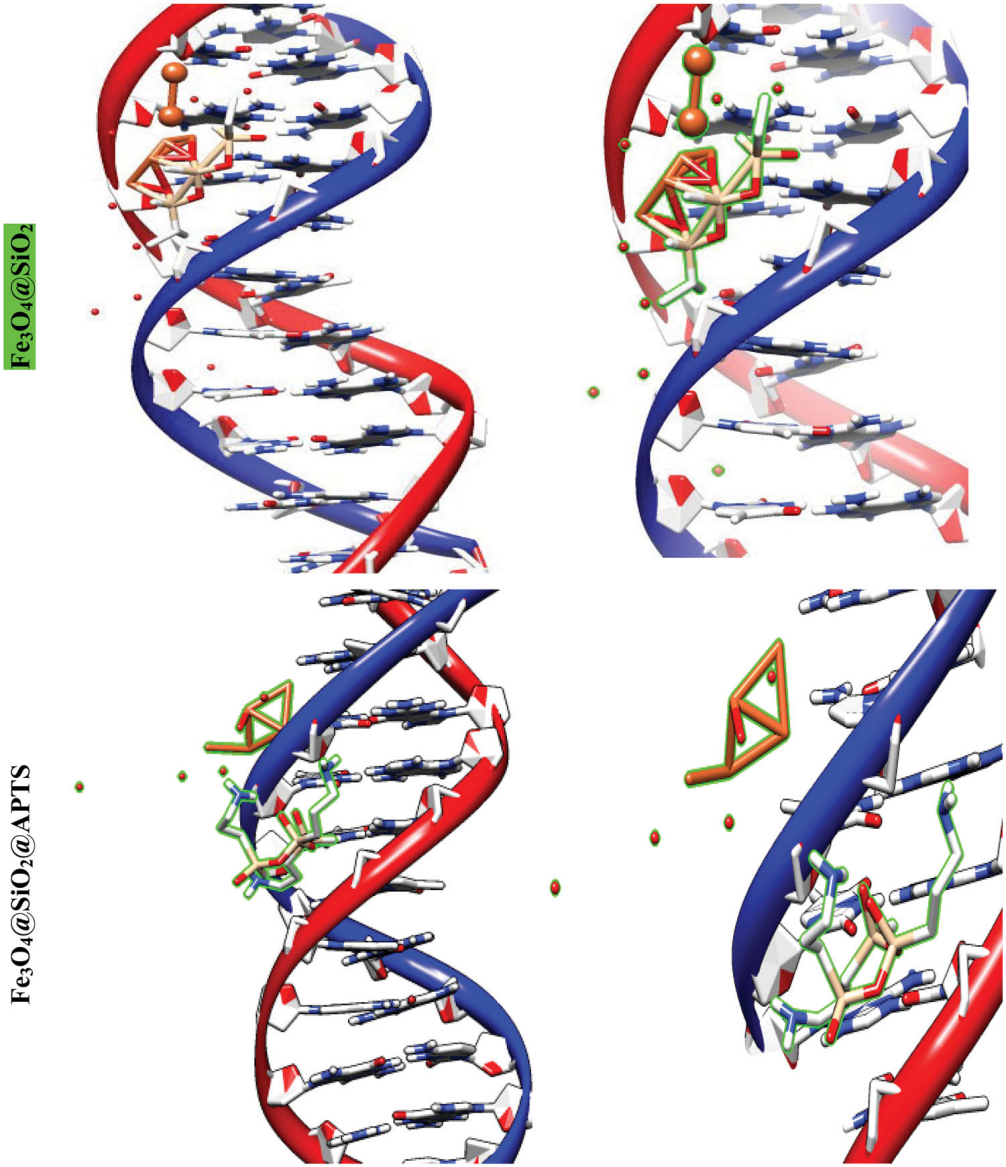

**Figure:**
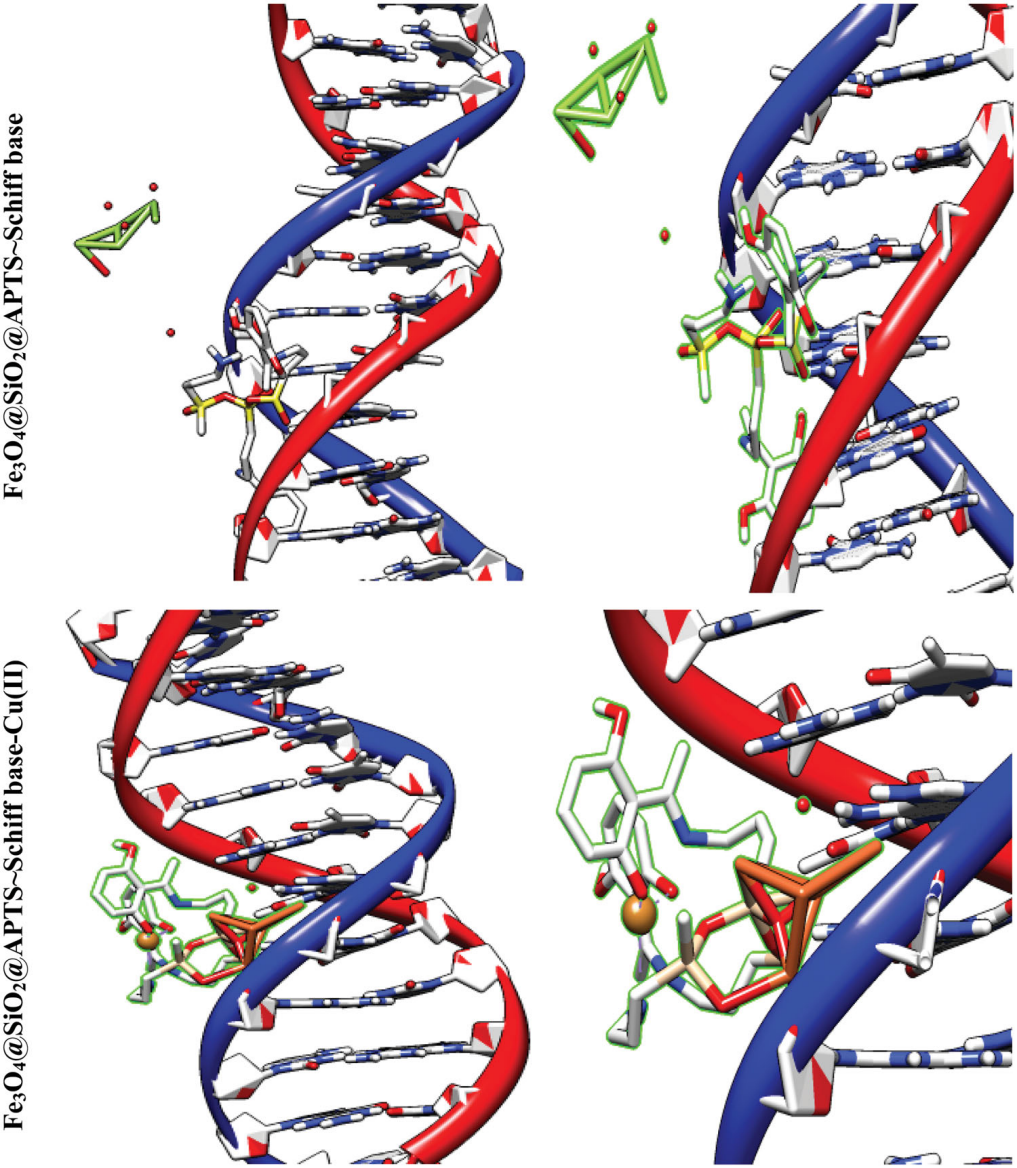

**Figure:**
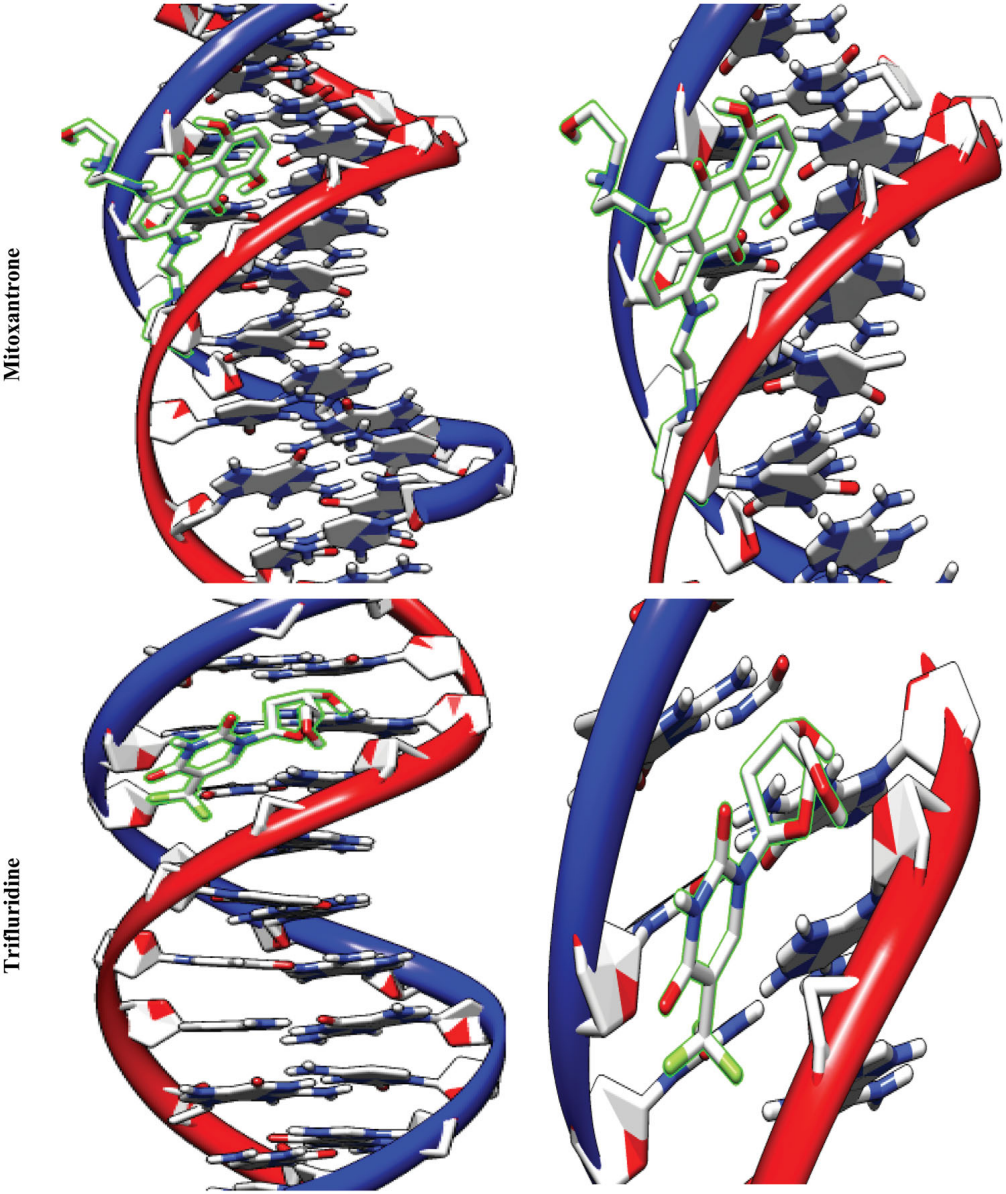

The docking results of compounds with topoisomerase II are shown in Supplementary Figure 9, listed in Table 3, the values of docking energy are −3.69, −6.94, −7.70, −3.52, and −7.94 kcal mol^−1^ for doxorubicin, Fe_3_O_4_@SiO_2_, Fe_3_O_4_@SiO_2_@APTS; Fe_3_O_4_@SiO_2_@APTS ∼ Schiff base and Fe_3_O_4_@SiO_2_@APTS ∼ Schiff base-Cu(II) docked to topoisomerase II, respectively. All compounds like doxorubicin binds directly to a DNA, but the binding into the DNA of Fe_3_O_4_@SiO_2_@APTS and Fe_3_O_4_@SiO_2_@APTS ∼ Schiff base-Cu(II) are higher than those of Fe_3_O_4_@SiO_2_@APTS ∼ Schiff base and doxorubicin. 3D pictures of docking conformations revealed that all the compounds were significantly inserted with DNA topoisomerase II via the major groove. Fe_3_O_4_@SiO_2_@APTS ∼ Schiff base-Cu(II) exhibited binding at Glu A:495, Ile A:658, Lys A:655, and Lys A:656 amino acid residue using hydrogen, metal acceptor, alkyl and pi-alkyl bonds. In addition, Fe_3_O_4_@SiO_2_@APTS ∼ Schiff base was docked with Met A:766 amino acid residue using Pi-sulfur, Fe_3_O_4_@SiO_2_@APTS docked with Lys A:798 and Ser A:763 using hydrogen, alkyl bonds and Fe_3_O_4_@SiO_2_ docked with Ser B:244, Lys A:243, ASN B:211, and ASN B:207 using hydrogen.

**Table 2.** DNA docking results of the compounds (unit: kcal/mol).

Structures	Estimated free energy of binding* (kcal/mol)	Final intermolecular energy (kcal/mol)	vdW + Hbond + desolv energy (kcal/mol)	Electrostatic energy (kcal/mol)	Final total internal energy (kcal/mol)	Torsional free energy (kcal/mol)	Unbound system's energy (kcal/mol)
Mitoxantrone	–10.35	–15.07	–11.84	–3.23	–4.48	+4.77	–4.48
Trifluridine	–5.56	–6.93	–6.85	–0.09	–0.82	+1.37	–0.82
Fe_3_O_4_@SiO_2_	–6.46	–12.42	–12.25	–0.17	–3.29	+5.97	–3.29
Fe_3_O_4_@SiO_2_@APTS	–10.85	–11.20	–10.39	–5.67	–1.58	+5.21	–1.58
Fe_3_O_4_@SiO_2_@APTS ∼ Schiff base	–7.47	–14.60	–13.30	–1.30	–4.68	+7.13	–4.68
Fe_3_O_4_@SiO_2_@APTS ∼ Schiff base-Cu(II)	–7.59	–8.69	–8.45	–0.24	–0.13	+1.10	–0.13

*ΔGbinding = ΔGvdW + hb+desolv + ΔGelec + ΔGtotal + ΔGtor − Δgunb

The docking results of compounds with ribonucleotide reductase are shown in Figure 6 and Tables 4a and 4b. Highest binding affinity of the drug has been identified based on the lowest docking energy. The binding free energy of the Fe_3_O_4_@SiO_2_, Fe_3_O_4_@SiO_2_@APTS, Fe_3_O_4_@SiO_2_@APTS ∼ Schiff base, Fe_3_O_4_@SiO_2_@APTS ∼ Schiff base-Cu(II), and triapine is dominated by −2.13, −8.45, −5.22, −7.21, and −3.03 kcal mol^−1^, respectively. In this simulation, Fe_3_O_4_@SiO_2_ is located in the pocket formed by Ser B:244, Met B:115, Gly B:214, and Lys B:243 (interaction with the ferric ions), as well as, Asn B:211, and Asn B:207 (interaction with the oxygen ions. Fe_3_O_4_@SiO_2_@APTS is located in the pocket formed by Ala B:263 (interaction with one of the ferric ions), Asp B:360, Glu B:361, Asn B:354, and Asp B:225 (three strong hydrogen bonds) and the other hydrophobic non-bonded interactions. Furthermore, Fe_3_O_4_@SiO_2_@APTS ∼ Schiff base is involved in hydrophobic non-bonded interactions (van der Waals bonding and carbon hydrogen bond). The interactions between Fe_3_O_4_@SiO_2_@APTS ∼ Schiff base-Cu(II) and the active sites on the target receptors may be non-covalent interactions like, hydrophobic, and van der Waals interaction. The conserved residues through Pi-sulfur (Met B:115), interaction with the ferric and copper ions (Leu B:157, Arg B:173, and Asn A:266), Glu115, hydrogen bonding (Ser B:154), metal acceptor interaction with one of the ferric ions (Tyr B:155) and van der Waals bonding lie around the pocket. At last, triapine had a strong binding affinity of Pi-sulfur toward Tyr A:568, attractive charge Asp A:569, three hydrogen bond formation with Thr A:574, Tyr A:568, Pro A:575, Lys A:582, and van der Waals bonding.

Figure 6.Docking conformation of all synthesized compounds and triapine with ribonucleotide reductase (3hne).

**Figure:**
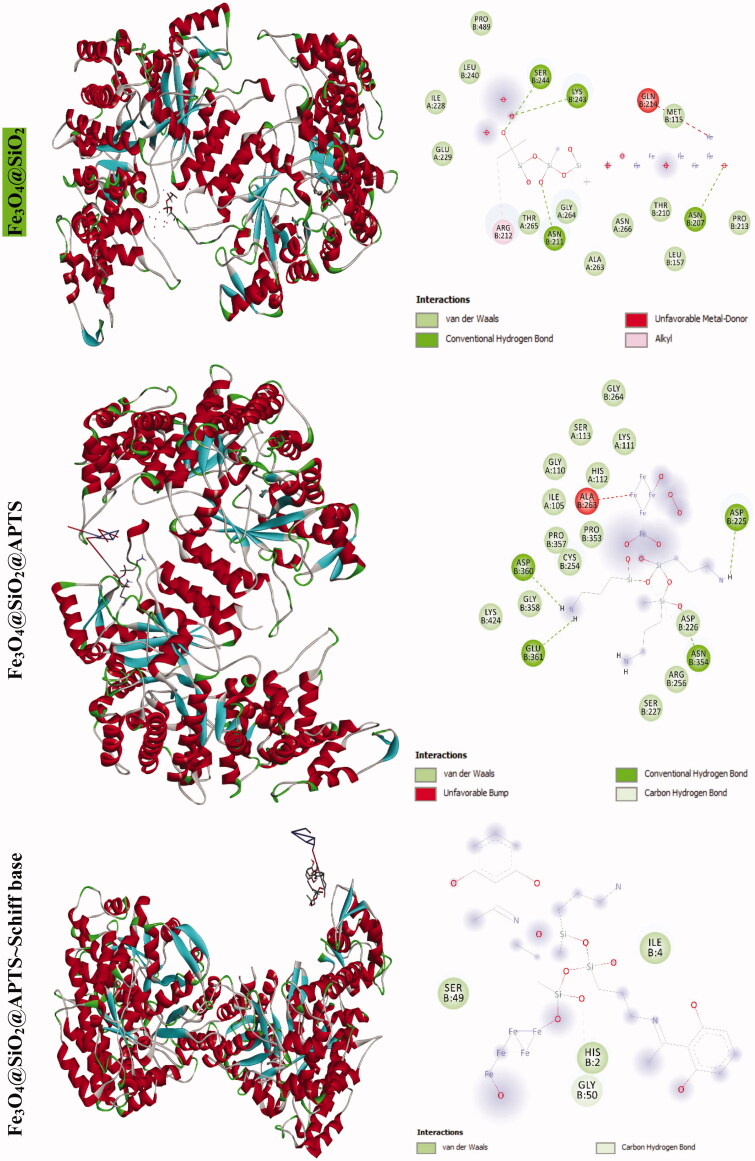

**Figure:**
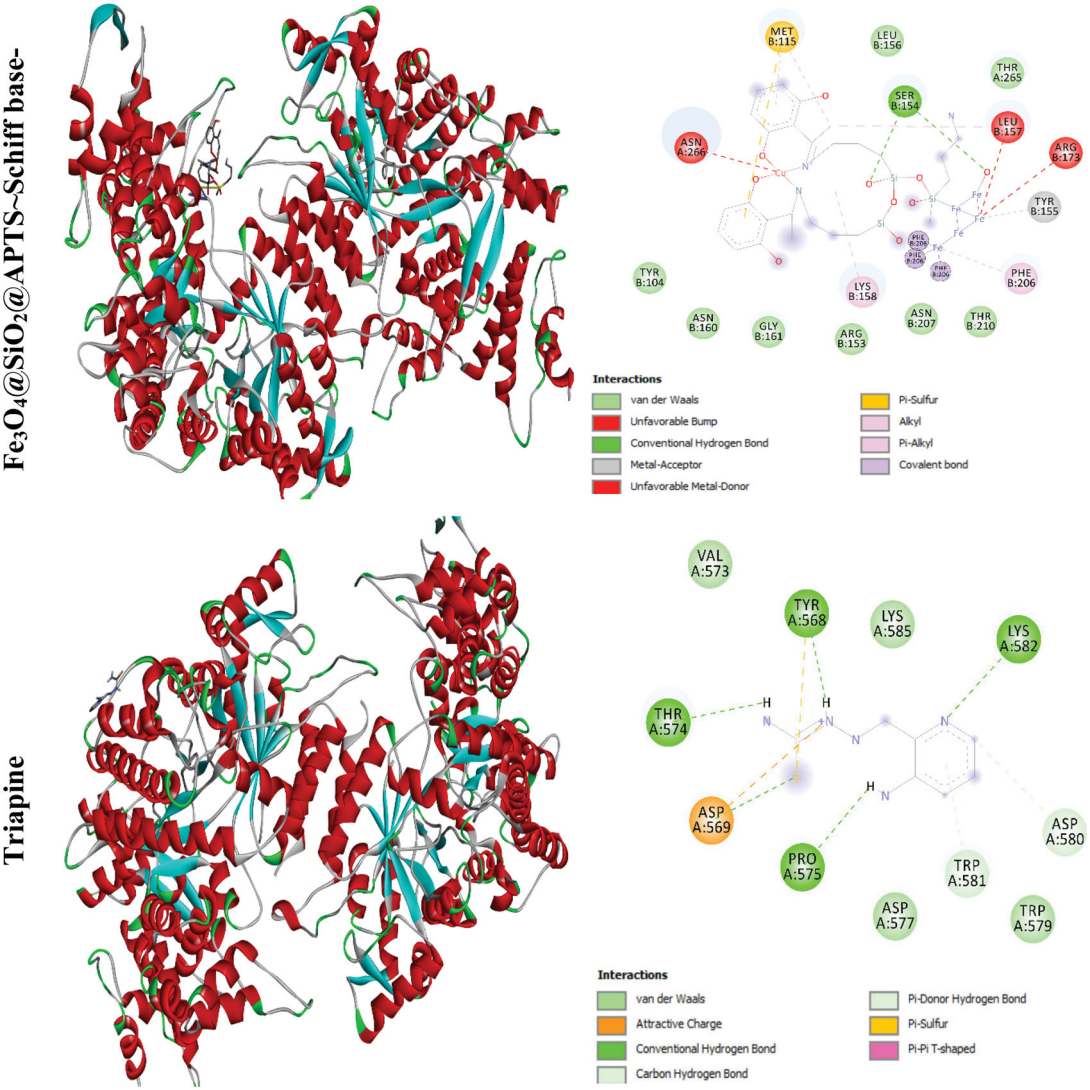

**Table 3.** Topoisomerase II docking results of the compounds (unit: kcal/mol).

Structures	Estimated free energy of binding* (kcal/mol)	Final intermolecular energy (kcal/mol)	vdW + Hbond + desolv energy (kcal/mol)	Electrostatic energy (kcal/mol)	Final total internal energy (kcal/mol)	Torsional free energy (kcal/mol)	Unbound system's energy (kcal/mol)
Doxorubicin	–6.94	–10.22	–8.37	+1.85	–4.88	+3.28	–4.88
Fe_3_O_4_@SiO_2_	–3.69	–9.65	–9.61	+0.05	–1.72	+5.97	–1.72
Fe_3_O_4_@SiO_2_@APTS	–7.70	–14.03	–7.52	–6.51	–2.45	+6.26	–2.45
Fe_3_O_4_@SiO_2_@APTS ∼ Schiff base	–3.52	–10.68	–7.78	–2.9	–5.82	+7.16	–5.82
Fe_3_O_4_@SiO_2_@APTS ∼ Schiff base-Cu(II)	–7.94	–11.22	–9.09	–2.13	–2.06	+3.28	–2.06

*ΔGbinding = ΔGvdW + hb+desolv + ΔGelec + ΔGtotal + ΔGtor − Δgunb

**Table 4a.** Ribonucleotide reductase docking results of compounds (unit: kcal/mol).

Structures	Estimated free energy of binding* (kcal/mol)	Final intermolecular energy (kcal/mol)	vdW + Hbond + desolv energy (kcal/mol)	Electrostatic energy (kcal/mol)	Final total internal energy (kcal/mol)	Torsional free energy (kcal/mol)	Unbound system's energy (kcal/mol)
Triapine	–3.03	–4.52	–4.45	–0.07	–0.89	+1.49	–0.89
Fe_3_O_4_@SiO_2_	–2.13	–8.10	–8.00	–0.10	–3.04	+5.97	–3.04
Fe_3_O_4_@SiO_2_@APTS	–8.45	–14.12	–8.06	–6.06	–1.37	+5.67	–1.37
Fe_3_O_4_@SiO_2_@APTS ∼ Schiff base	–5.22	–2.24	–1.81	–0.43	–14.22	+7.46	–14.22
Fe_3_O_4_@SiO_2_@APTS ∼ Schiff base-Cu(II)	–7.21	–8.40	–8.38	–0.02	–0.11	+1.19	–0.11

*ΔGbinding = ΔGvdW + hb+desolv + ΔGelec + ΔGtotal + ΔGtor − Δgunb

Phospholipids as bilayer are the major components of biological membranes and consists of an amphipathic (or amphiphilic) including hydrophilic (a polar head group) headgroup and a hydrophobic tail along with one or more cis-double bonds (two hydrophobic hydrocarbon tails). The structures of palmitoyloleoylphatidylcholine (POPC) were constituted by choline group. By analyzing the energy minimization of each optimized complexation state with POPC, results show the flexible docking algorithm (Cai et al., 2006).

**Figure 7.**
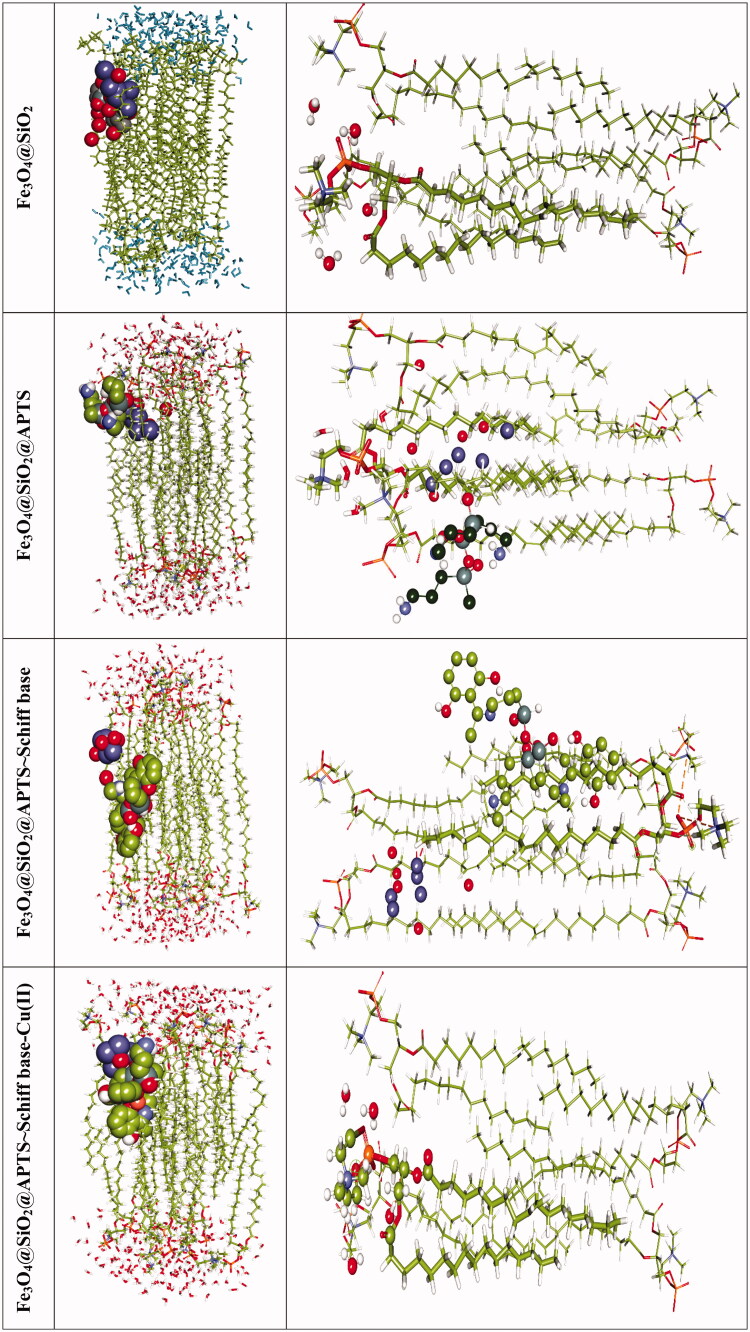
Docking conformation and interactions of all synthesized compounds with anionic membrane POPC.

The favorable binding energy minimization of the Fe_3_O_4_@SiO_2_, Fe_3_O_4_@SiO_2_@APTS, Fe_3_O_4_@SiO_2_@APTS ∼ Schiff base, Fe_3_O_4_@SiO_2_@APTS ∼ Schiff base-Cu(II) with POPC is dominated by −1.66, −3.31, −0.57, and −5.30 kcal mol^−1^, respectively. Our results indicated Fe_3_O_4_@SiO_2_@APTS ∼ Schiff base-Cu(II)>Fe_3_O_4_@SiO_2_@APTS > Fe_3_O_4_@SiO_2_>Fe_3_O_4_@SiO_2_@APTS ∼ Schiff base. From Figure 7, it can be seen that the electrostatic interaction of Fe_3_O_4_@SiO_2_@APTS and Fe_3_O_4_@SiO_2_@APTS ∼ Schiff base-Cu(II) is much stronger than Fe_3_O_4_@SiO_2_@APTS ∼ Schiff base and Fe_3_O_4_@SiO_2_. It is mainly due to the strong electrostatic interaction Fe_3_O_4_@SiO_2_@APTS and Fe_3_O_4_@SiO_2_@APTS ∼ Schiff base-Cu(II) with the acyl of polar group on the POPC headgroup. Meanwhile, Fe_3_O_4_@SiO_2_ and Fe_3_O_4_@SiO_2_@APTS ∼ Schiff base linked the hydrophobic tail of the phospholipid.

## 6. Discussion

In summary, the present work illustrates the importance of the molecular dynamic simulations of a series of compounds with grafting 2,4‐toluene diisocyanate as a bi‐functional covalent linker onto a nano-Fe_3_O_4_ support for first time. All compounds were characterized by FT‐IR, XRD, SEM, EDX, and VSM. The understanding of relationship between iron ions release and immunotoxicity of IONPs is important (Singh et al., 2010; Mahon et al., 2012). The IONPs are permeabilized through passive diffusion, clathrin-mediated endocytosis, caveolin-mediated internalization, and other clathrin and caveolin-independent endocytosis (Supplementary Figure 10). The toxicity activities of nanoparticles decreased through increasing nanoparticles chain length and additional protective that created low release of iron ions in intra-cellular space. As a result, additional protection enhances coated iron oxide NPs resistance to the lysosomal acidity, consequently reduction of the iron ions (Fe^+2^) release. The cell death is caused by interaction between Fe^+2^ with hydrogen peroxide to produce highly reactive hydroxyl radicals and the Fenton reaction in the mitochondria to produce ferric ions (Fe^3+^) (Halliwell & Gutteridge, 2015). The ROS generation does not counterbalance the action of antioxidant enzymes and may damage biomolecules such as DNA, lipids, and proteins (Palmieri & Sblendorio, 2007; Birben et al., 2012; Abdesselem et al., 2017). Positively charged IONPs generated more ROS compared to neutral and negatively charged IONPs due to strong electrostatic interaction between the negatively charged cell surface and positively charged IONPs (Cai et al., 2013). Amine-modified IONPs were found to be more lethal *in vitro* tests (Chang et al., 2012; Shen et al., 2012).

**Table 4b.** Lipid docking results of compounds (unit: kcal/mol).

Structures	Estimated free energy of binding* (kcal/mol)	Final intermolecular energy (kcal/mol)	vdW + Hbond + desolv energy (kcal/mol)	Electrostatic energy (kcal/mol)	Final total internal energy (kcal/mol)	Torsional free energy (kcal/mol)	Unbound system's energy (kcal/mol)
Fe_3_O_4_@SiO_2_	–1.66	–7.62	–7.62	–0.01	–3.17	+5.97	–3.17
Fe_3_O_4_@SiO_2_@APTS	–3.31	–9.87	–8.64	–1.24	–4.04	+6.56	–4.04
Fe_3_O_4_@SiO_2_@APTS ∼ Schiff base	–0.57	–10.11	–10.09	–0.02	–7.25	+9.55	–7.25
Fe_3_O_4_@SiO_2_@APTS ∼ Schiff base-Cu(II)	–5.30	–9.18	–8.81	–0.37	–1.82	+3.88	–1.82

*ΔGbinding = ΔGvdW + hb+desolv + ΔGelec + ΔGtotal + ΔGtor − Δgunb

Bona et al. synthesized prepared the hydrophilic ligands polyethyleneimine or poly(acrylic acid) on the surface of the NPs and investigated *in vivo*. The results showed bioaccumulation and toxicity with a positively charged surface coating greater than nanoparticles with positively charged surface coating in the fetus (Di Bona et al., 2014).

Fe_3_O_4_@SiO_2_@APTS ∼ Schiff base-Cu(II) demonstrated a higher anticancer properties than Fe_3_O_4_@SiO_2_@APTS ∼ Schiff base, Fe_3_O_4_@SiO_2_@APTS, and Fe_3_O_4_@SiO_2_ (Dunford, 2002; Voinov et al., 2011). Studies on the cytotoxicity properties of these compounds demonstrated lesser toxicity effects at doses of below 0.01 mg/mL (Karlsson et al., 2008; Ankamwar et al., 2010; Malvindi et al., 2014). Due to nano-sized, they might surpass blood–brain barrier (BBB) as protective barrier and damage neural functions and central nervous system (CNS), also can cross nuclear membrane and result mutations (Malhotra & Prakash, 2011; Gholami et al., 2015). Uncoated iron oxide NPs have very low solubility which can lead to agglomeration in lungs and liver under physiological conditions and can impede blood vessels and rapidly remove macrophages leading to thromboses formation. Hence, to improve biocompatibility, dispersibility and bio-distribution, nanoparticles are coated with silica, dextran, citrate, and PEGylated starch. Functional groups of coated iron oxide NPs interact with relevant ligands and polymers to play cytotoxicity. The creation of organic molecules on iron oxide NPs surface is a fundamental step to improve structures: (i) increase stability in a pH around 7.4 and (ii) reduce adverse cellular effects. Nowadays, superparamagnetic iron oxide nanoparticles are already used medical treatments against cancer diseases and their lower systemic adverse effects were demonstrated for a long time in the human body (Hussain et al., 2005; Kim et al., 2006).

Molecular docking studies along with experimental studies could help to explore a potential drug candidate. Molecular docking simulation was carried out for all compounds with DNA, ribonucleotide reductase, and topoisomerase II. Development of novel transition-metal-based drugs which bind DNA by noncovalent modes including major and minor groove binding, electrostatic effect between the negatively charged nucleic sugar–phosphate backbone and the positive or negative end of the compounds, and intercalation is priority (Yang et al., 2017; Salehi et al., 2019). DNA topoisomerase II inhibitors such as the anthracyclines (daunorubicin and doxorubicin) bind to the transient enzyme–DNA complex and inhibit the activity of DNA Topo2 enzyme and DNA replication (Arthur, 2019). The enzyme ribonucleotide reductase as ubiquitous cytosolic enzyme catalyzes the DNA and it is one of the most important target for cancer therapy and antiviral agents in DNA synthesis, growth, metastasis, and drug resistance of cancer cells (Zaltariov et al., 2017).

The molecular docking studies of compounds showed that Fe_3_O_4_@SiO_2_@APTS strongly binds through minor groove with DNA by electrostatic and hydrogen energy (kcal/mol). In addition, the results show that the compounds Fe_3_O_4_@SiO_2_@APTS and Fe_3_O_4_@SiO_2_@APTS ∼ Schiff base-Cu(II) bind strongly into topoisomerase II compared to the Fe_3_O_4_@SiO_2_@APTS ∼ Schiff base and doxorubicin. The next stage, Fe_3_O_4_@SiO_2_@APTS and Fe_3_O_4_@SiO_2_@APTS ∼ Schiff base-Cu(II) bind strongly into ribonucleotide reductase compared to the Fe_3_O_4_@SiO_2_@APTS ∼ Schiff base and triapine. At last, Fe_3_O_4_@SiO_2_@APTS ∼ Schiff base-Cu(II) binds strongly into phospholipids compared to another compounds. Over all, metal complexes as the potential anticancer drug candidates exhibited capable of binding/cleaving DNA and proteins (Gupta et al., 2013).

## Supplementary Material

Supplemental Material
