# Supplementary material for: Synthesis and toxicity assessment of Fe3O4 NPs grafted by ∼ NH2-Schiff base as anticancer drug: modeling and proposed molecular mechanism through docking and molecular dynamic simulation
Source: Drug Deliv. 2020 Aug 10;27(1):1201–17. doi: 10.1080/10717544.2020.1801890 (PMC7470030; doi:10.1080/10717544.2020.1801890)
Supplement: Supplemental Material [file IDRD_A_1801890_SM7728.docx]

**Supplementary material**

**Synthesis and Toxicity Assessment of Fe_3_O_4_ NPs grafted by ~NH_2_-Schiff base as Anticancer Drug: Modeling and Proposed Molecular Mechanism through Docking and Molecular Dynamic Simulation**

Rahime Eshaghi Malekshah^a^, Bahareh Fahimirad^a^, Mohammadreza Aallaei^b^,

Ali Khaleghian^c,^*^^[[1]](#footnote-1)^^

*^a^Department of Chemistry, College of Science, Semnan University, Semnan, Iran*

*^b^Department of Chemistry, Faculty of Science, Imam Hossein University, Tehran, Iran*

*^c^Biochemistry Department, Faculty of Medicine, Semnan University of Medical Sciences, Semnan, Iran*

**Supplementary. Figure 1.** XRD patterns of Fe_3_O_4_@SiO_2_ (a) and Fe_3_O_4_@SiO_2_@APTS~Schiff base-Cu(II) (b).

**Supplementary. Figure 2.** VSM of Fe_3_O_4_@SiO_2_ (a) and Fe_3_O_4_@SiO_2_@APTS~Schiff base-Cu(II) (b).

| 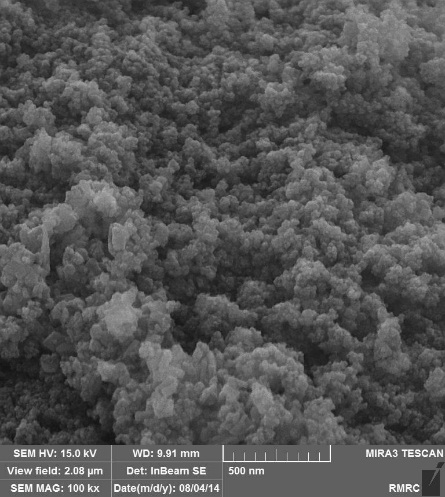 |  | **** |
| --- | --- | --- |
| (a) | (b) | (c) |

**Supplementary. Figure 3.** FE-SEM images of Fe_3_O_4_ (a); Fe_3_O_4_@SiO_2_@APTS~Schiff base-Cu(II) nanoparticles (b and c).


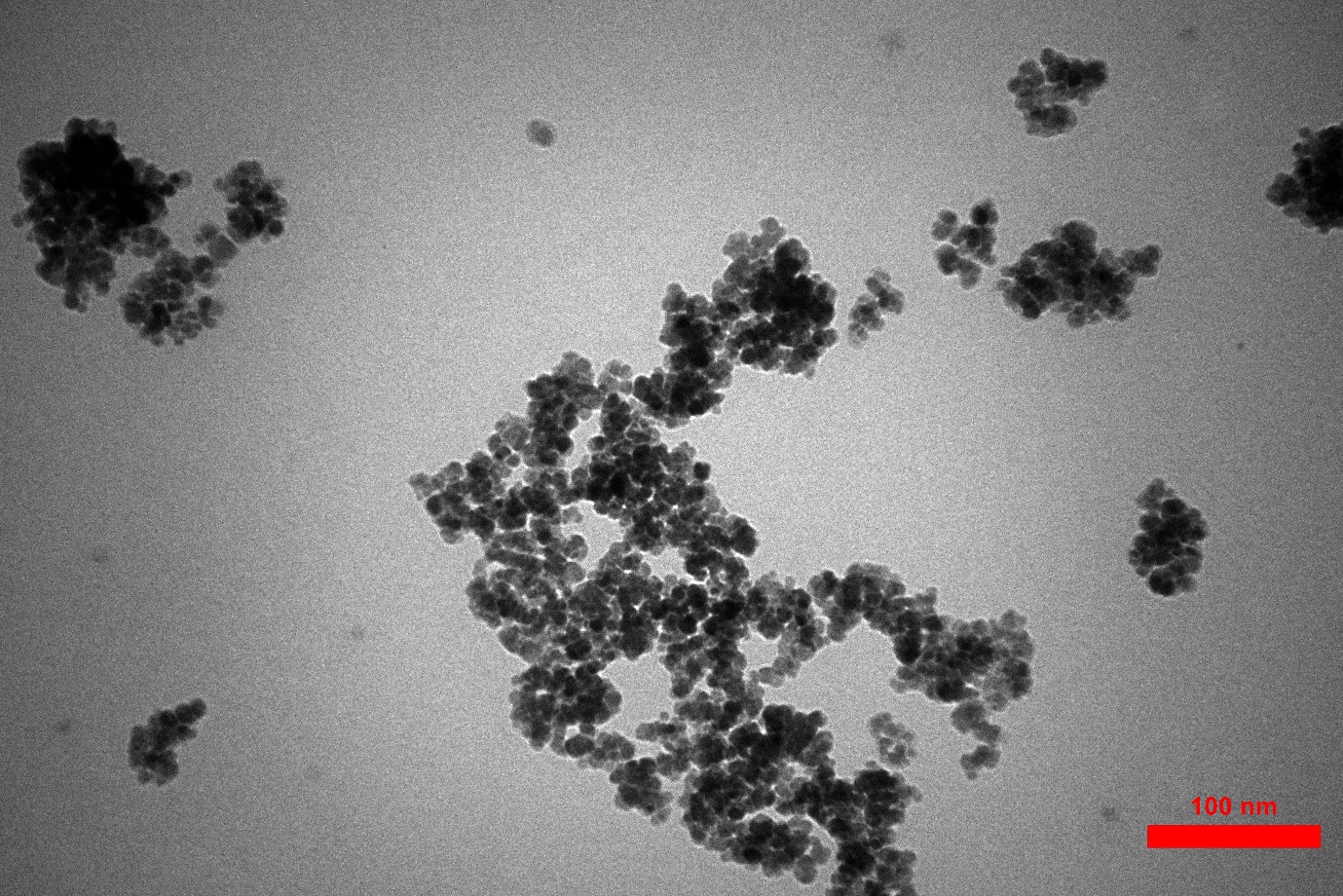


**Supplementary. Figure 4.** TEM images of Fe_3_O_4_@SiO_2_@APTS~Schiff base-Cu(II) nanoparticles

| **Fe_3_O_4_@SiO_2_@APTS** | 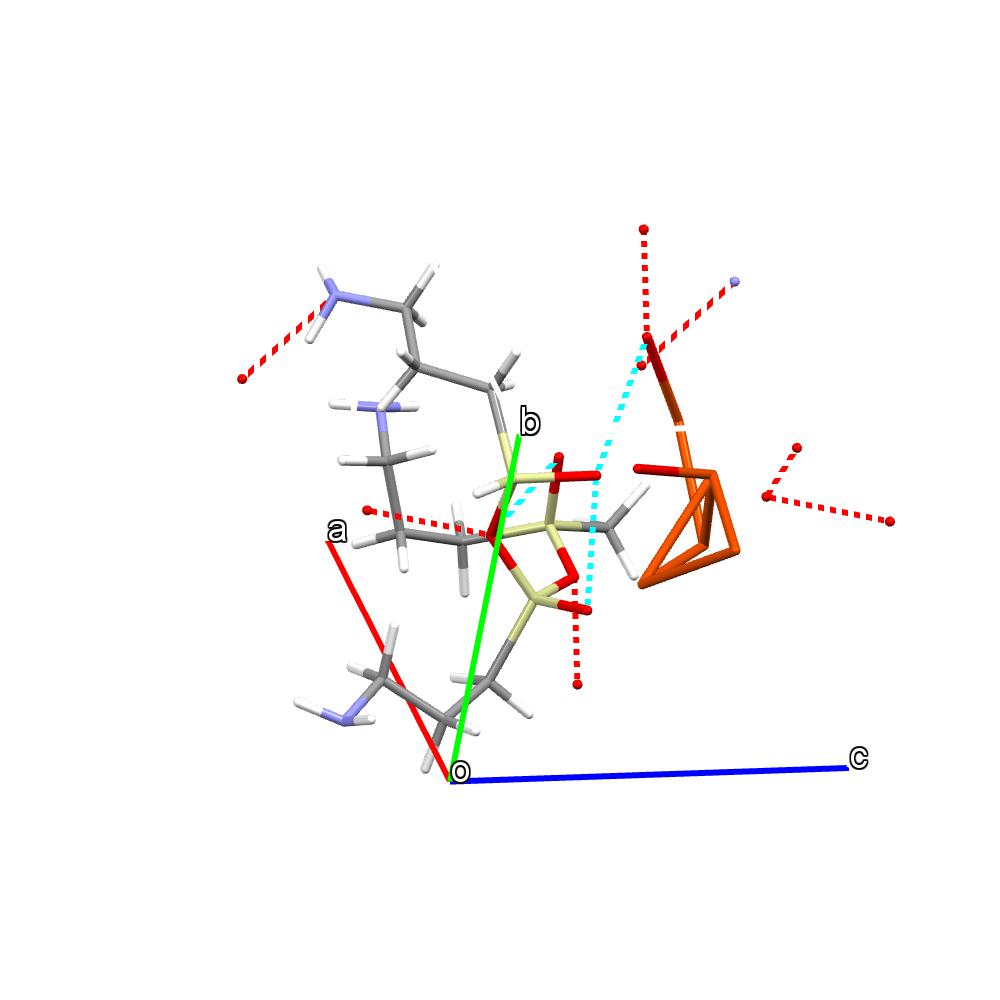 |
| --- | --- |
| **Fe_3_O_4_@SiO_2_@APTS~Schiff base** | 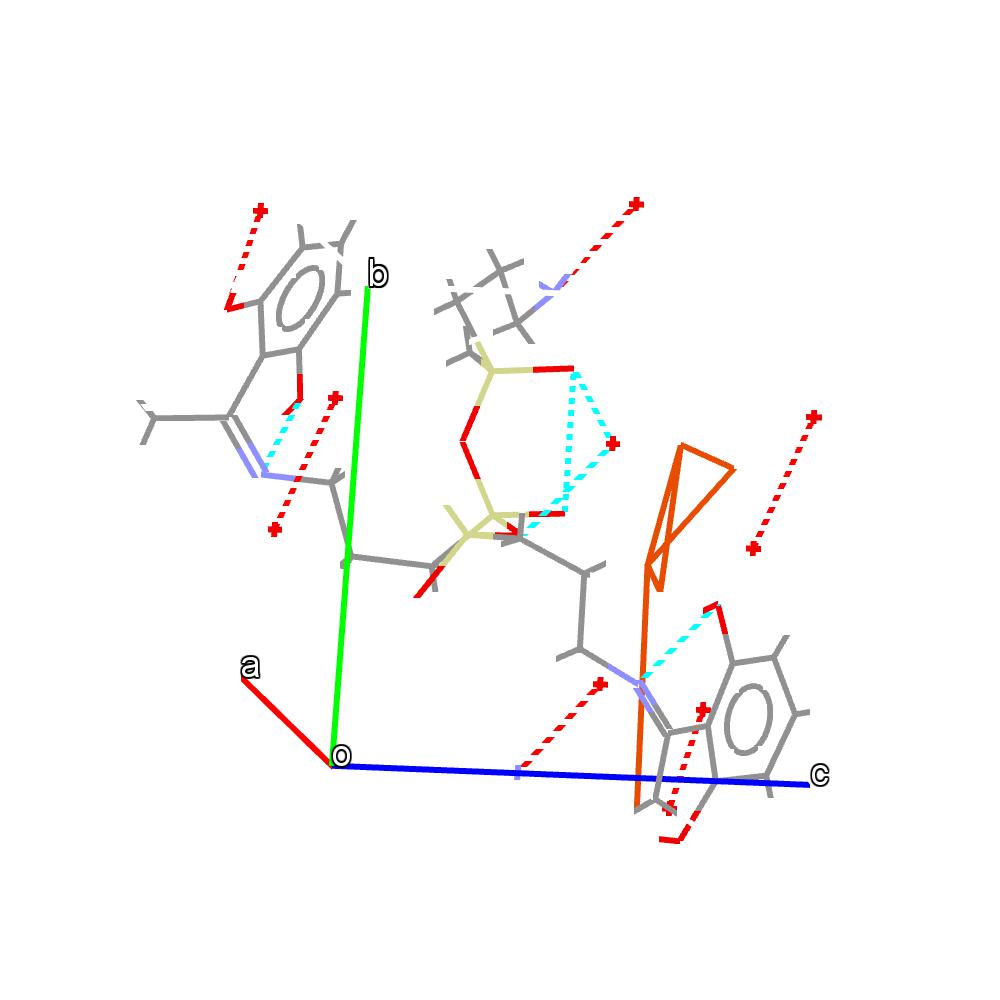 |
| **Fe_3_O_4_@SiO_2_@APTS~Schiff base-Cu(II)** | 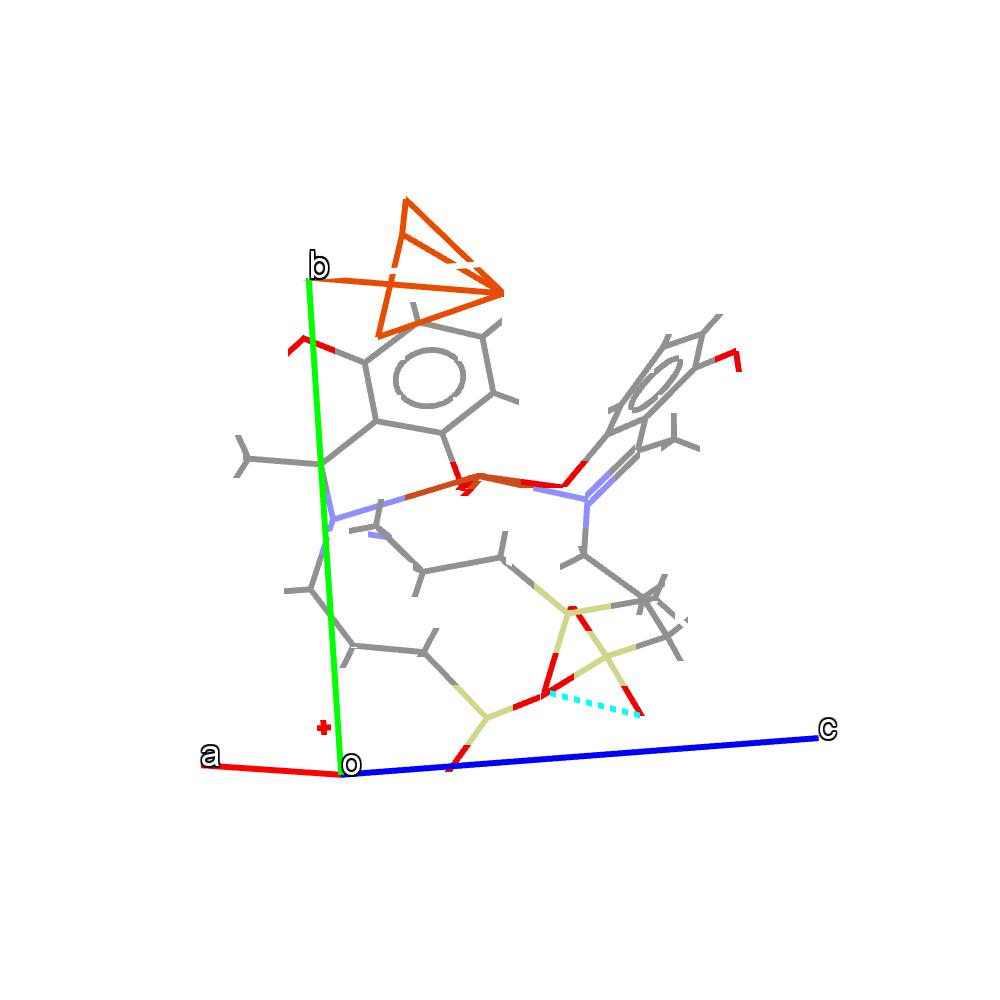 |
| **Supplementary. Fig 5.** Intermolecular and π–π stacking interactions in the structures of Fe_3_O_4_@SiO_2_@APTS, Fe_3_O_4_@SiO_2_@APTS~Schiff base and Fe_3_O_4_@SiO_2_@APTS~Schiff base-Cu(II) | |

| **Fe_3_O_4_@SiO_2_** | 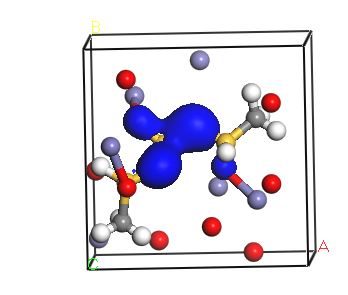 | 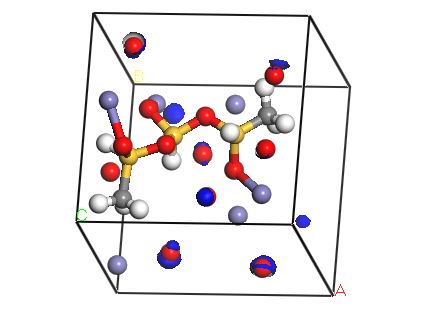 |
| --- | --- | --- |
| **Fe_3_O_4_@SiO_2_@APTS** | 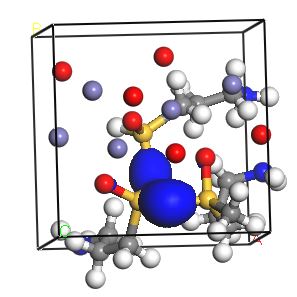 | 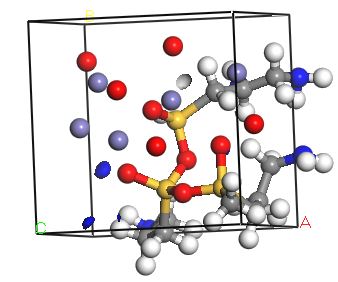 |
| **Fe_3_O_4_@SiO_2_@APTS~Schiff base** | 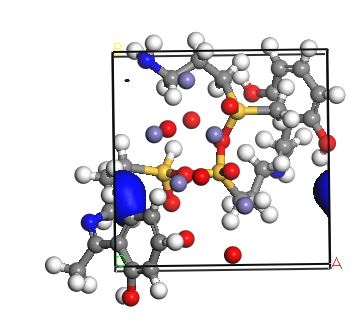 | 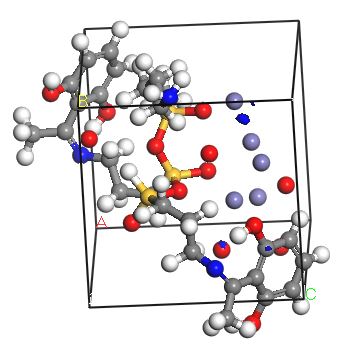 |
| **Fe_3_O_4_@SiO_2_@APTS~Schiff base-Cu(II)** | 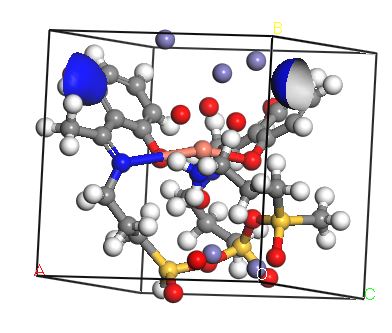 | 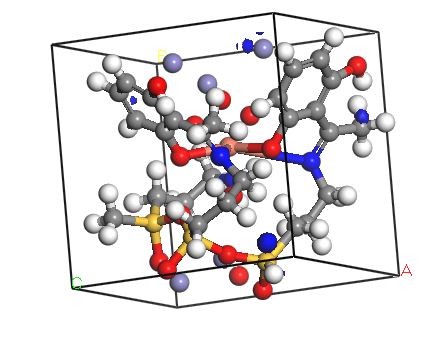 |
|  | HOMO | LUMO |
| **Supplementary. Fig 6.** Frontier orbital density distributions (HOMO and LUMO) for Fe_3_O_4_@SiO_2,_ Fe_3_O_4_@SiO_2_@APTS, Fe_3_O_4_@SiO_2_@APTS~Schiff base and Fe_3_O_4_@SiO_2_@APTS~Schiff base-Cu(II) in aqueous phase.   \| **Fe_3_O_4_@SiO_2_** \| 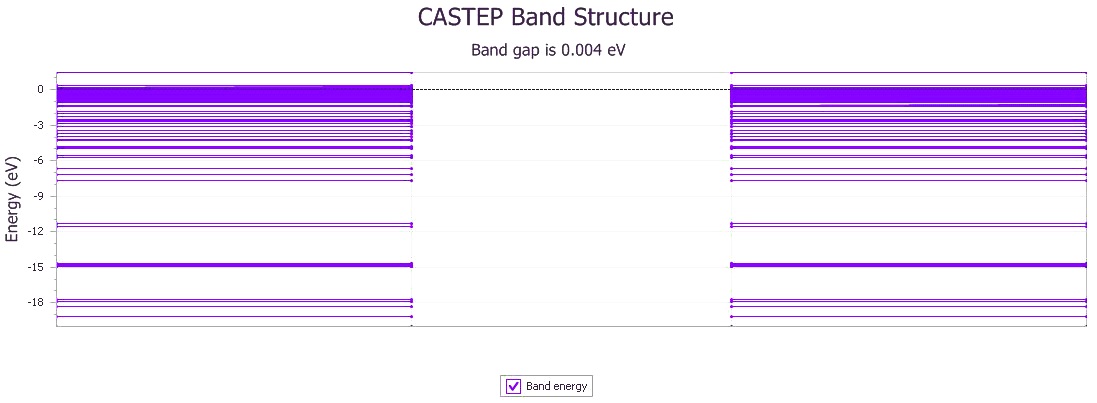 \| \| --- \| --- \| \| **Fe_3_O_4_@SiO_2_@APTS** \| 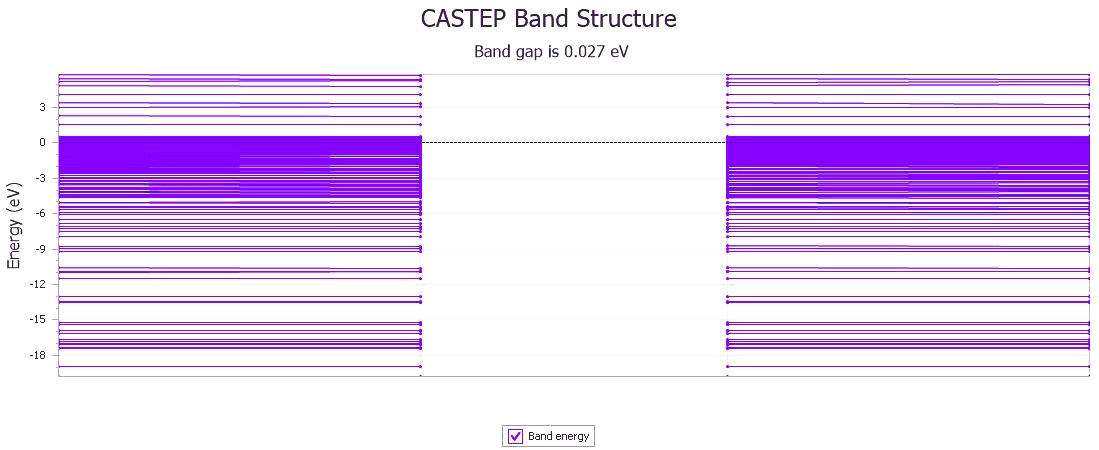 \| \| **Fe_3_O_4_@SiO_2_@APTS~Schiff base** \| 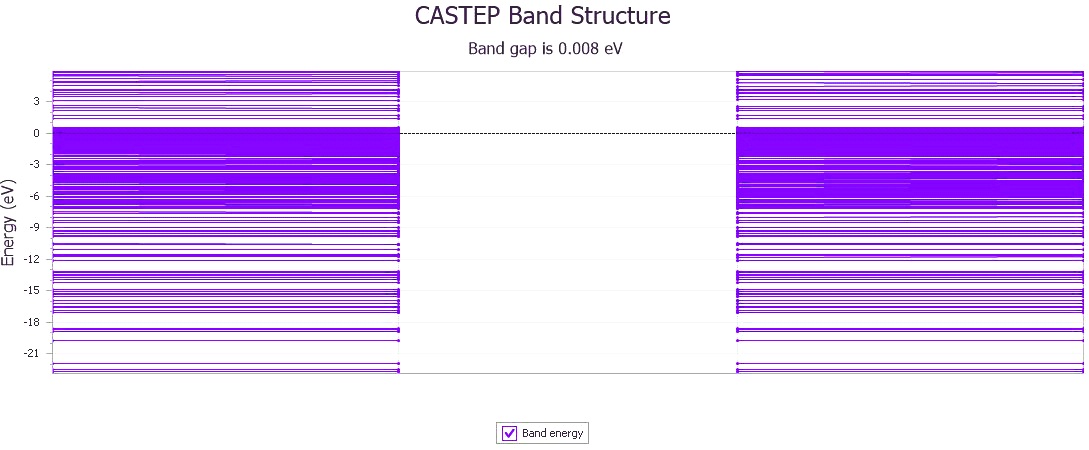 \| \| **Fe_3_O_4_@SiO_2_@APTS~Schiff base-Cu(II)** \| 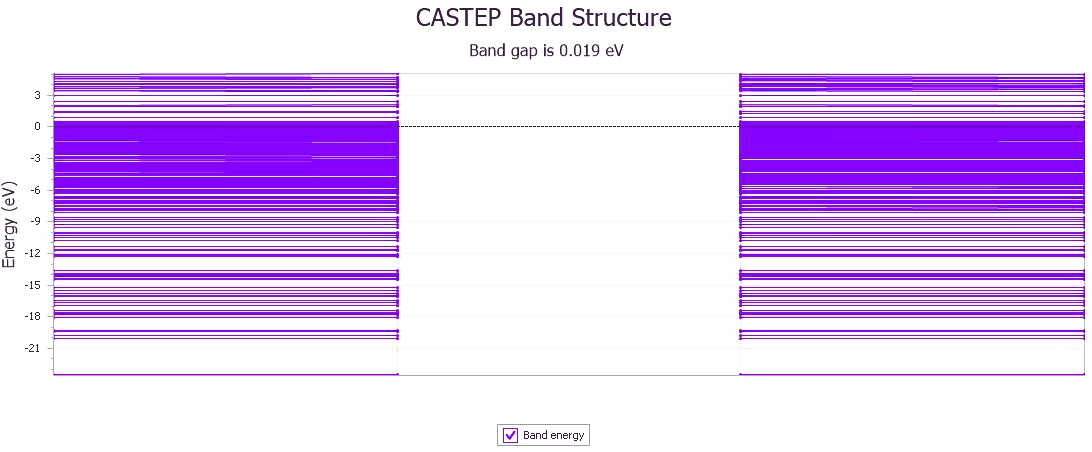 \| \| **Supplementary. Fig 7.** CASTEP Band Structure Band Gap \| \| | | |

| 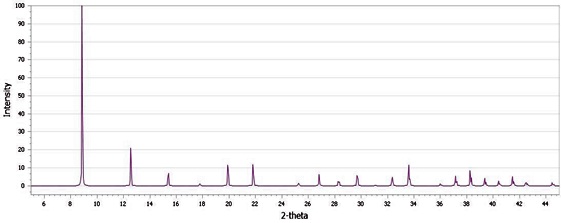 |
| --- |
| **Supplementary. Fig 8.** The optimized computational XRD of Fe_3_O_4_@SiO_2_@APTS~Schiff base-Cu(II). |

| **Fe_3_O_4_@SiO_2_** | 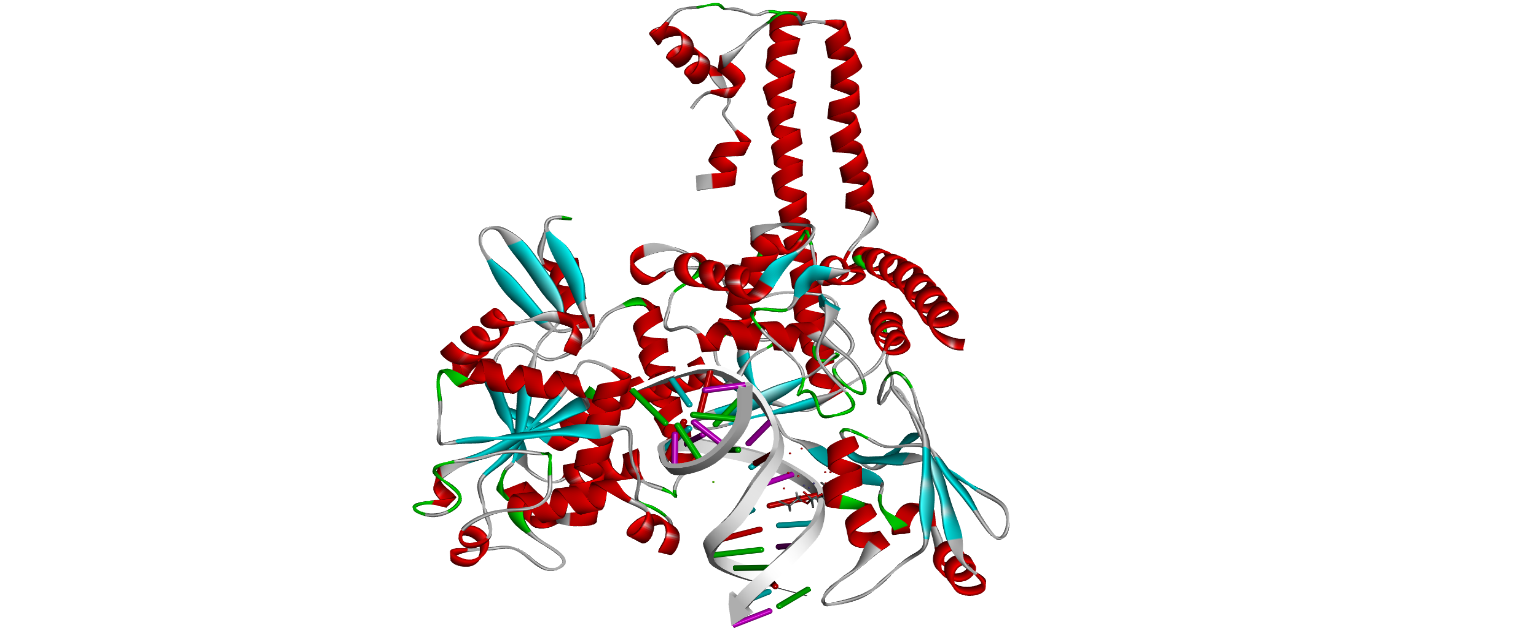 | 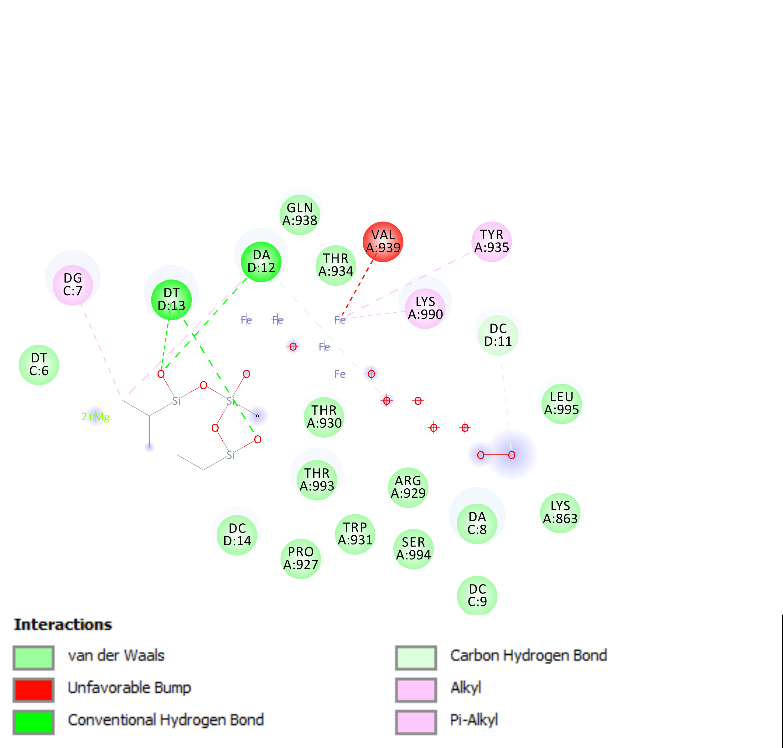 |
| --- | --- | --- |
| **Fe_3_O_4_@SiO_2_@APTS** | 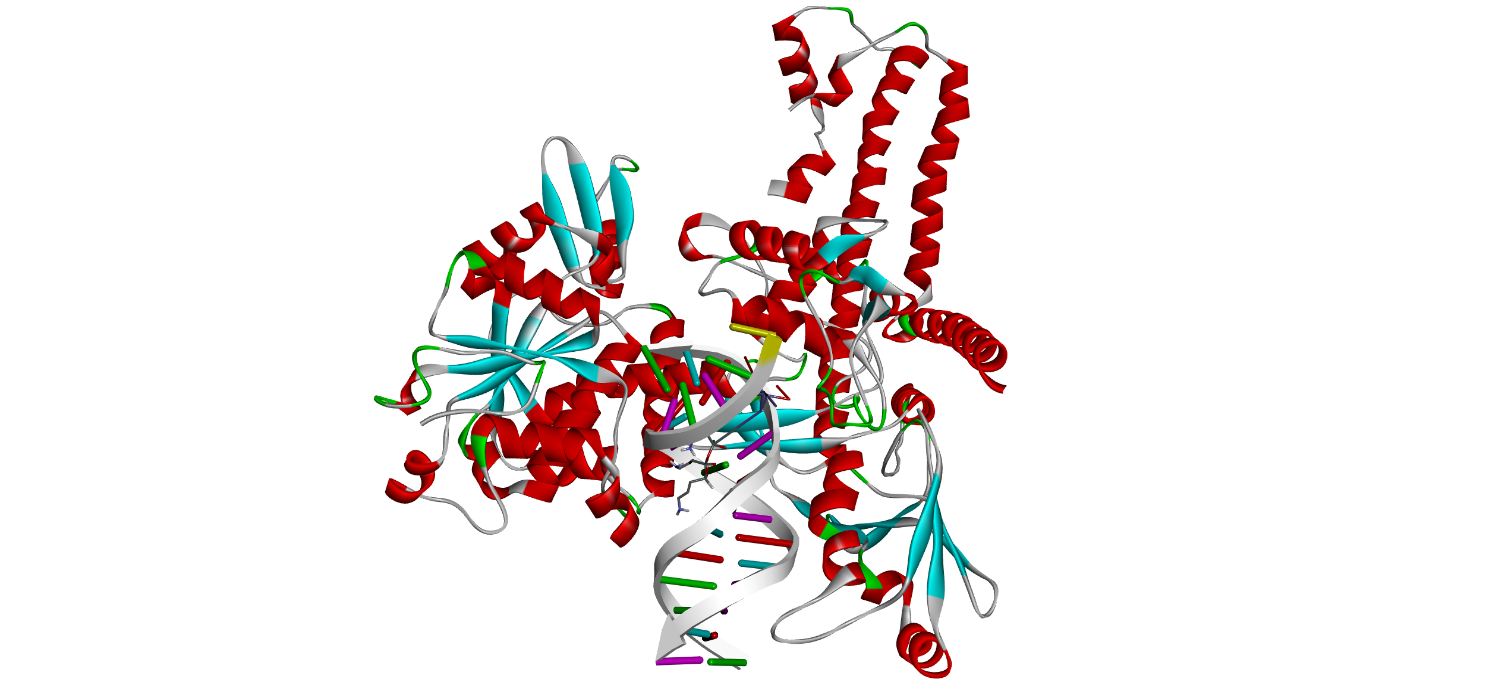 | 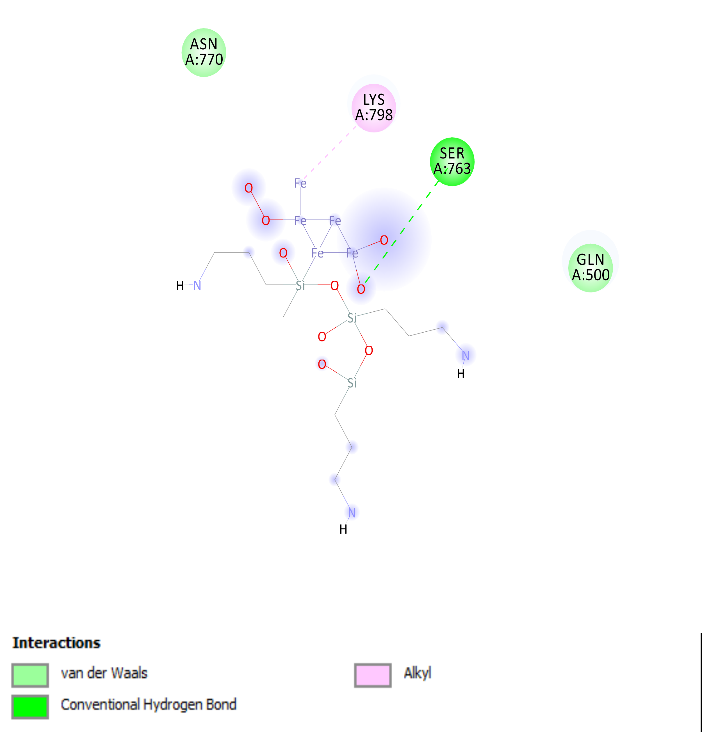 |
| **Fe_3_O_4_@SiO_2_@APTS~Schiff base** | 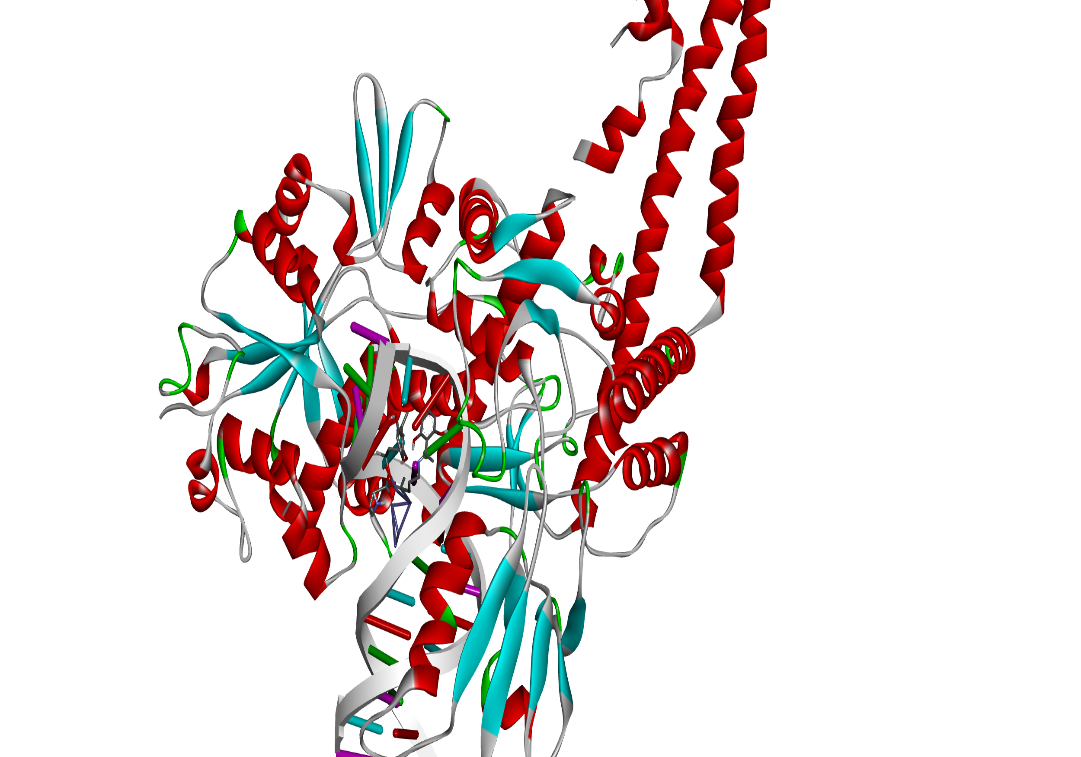 | 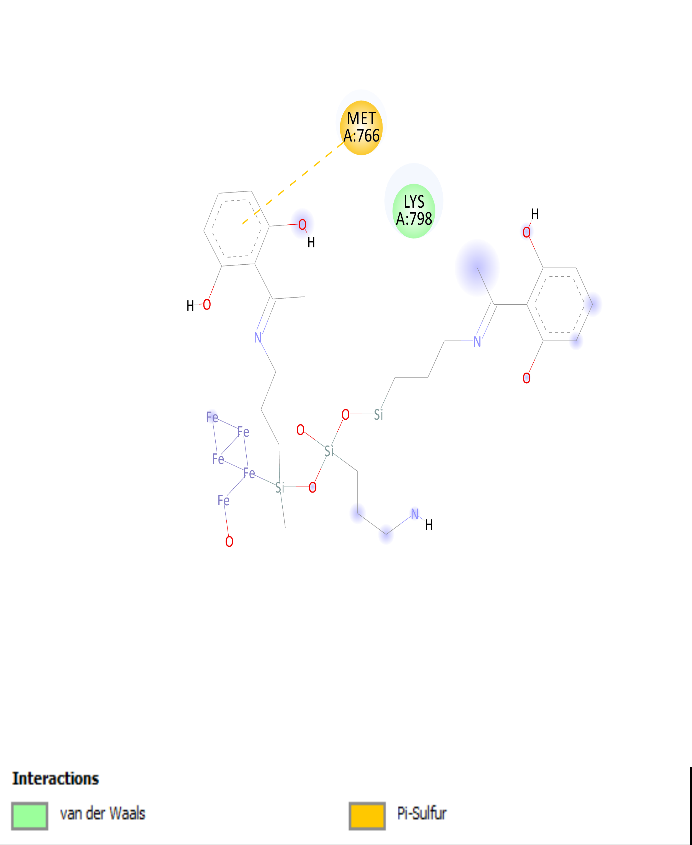 |
| **Fe_3_O_4_@SiO_2_@APTS~Schiff base-Cu(II)** | 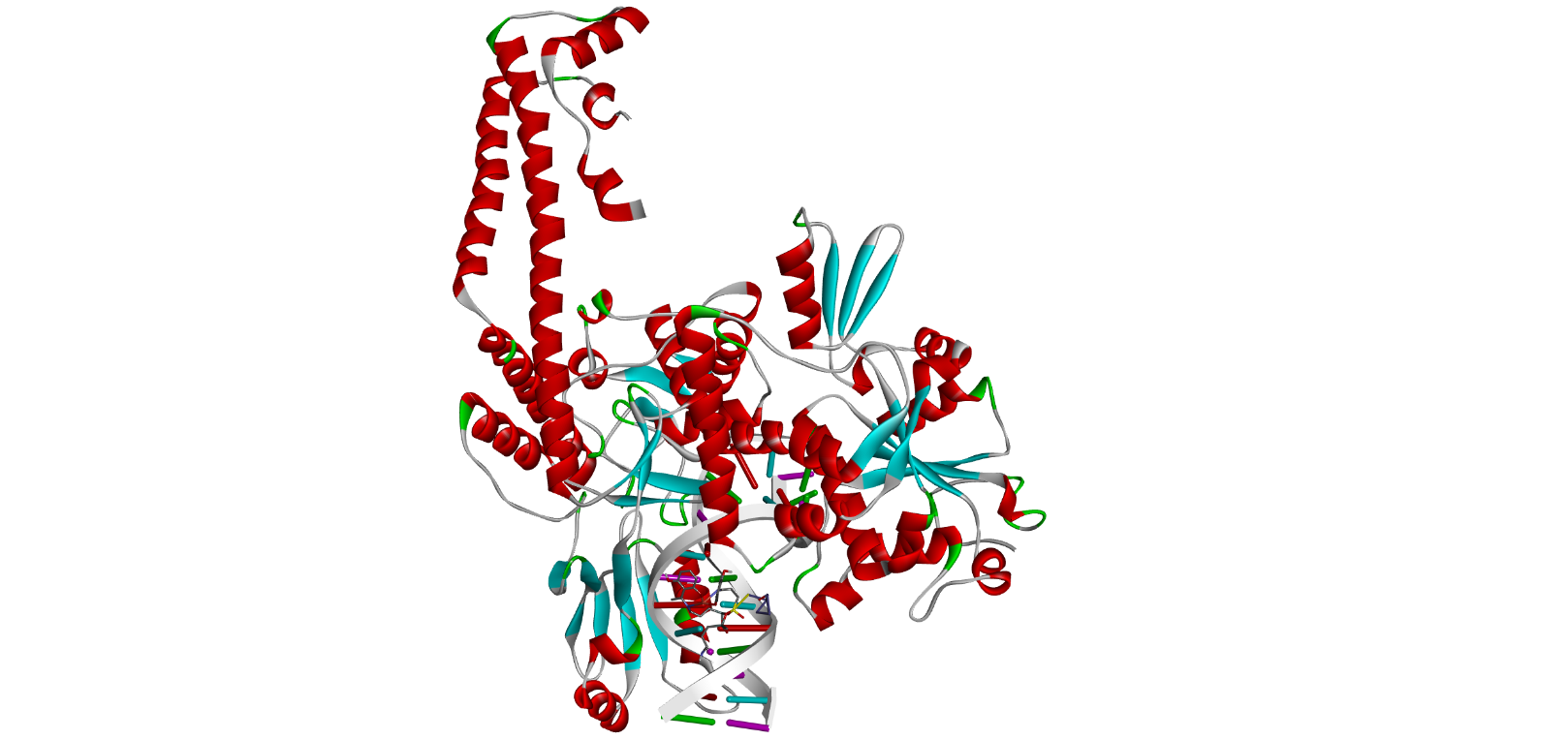 | 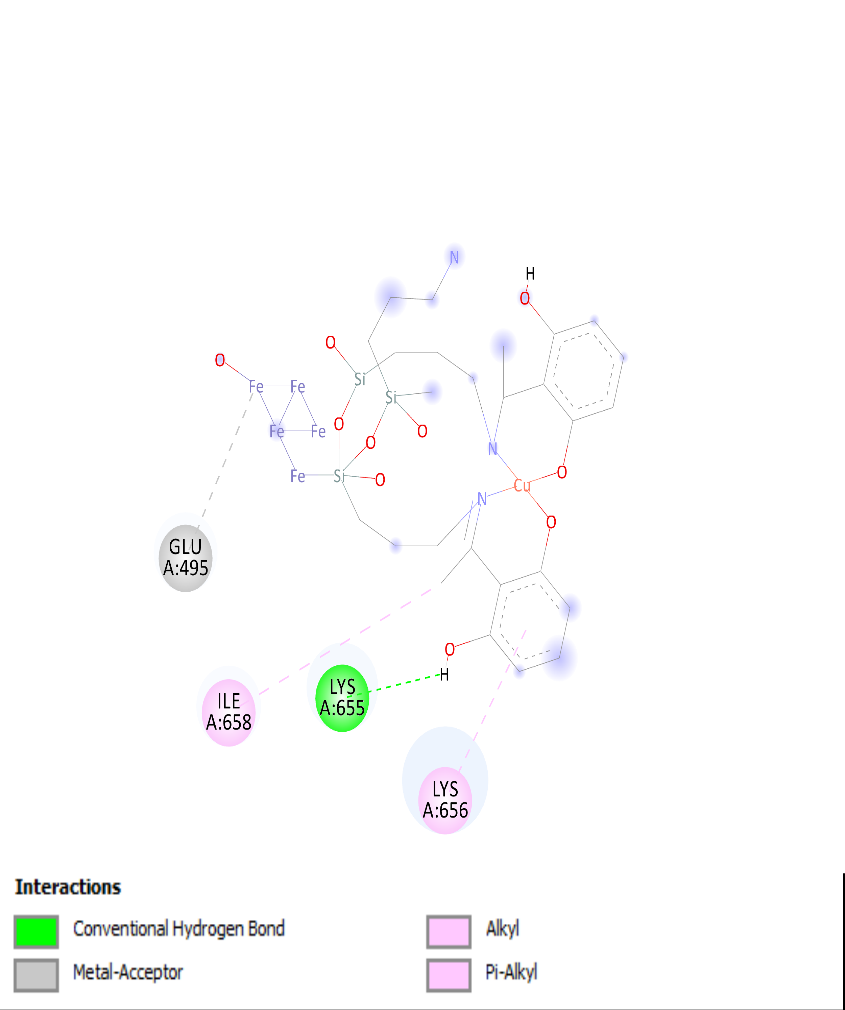 |
| **Doxorubicin** | 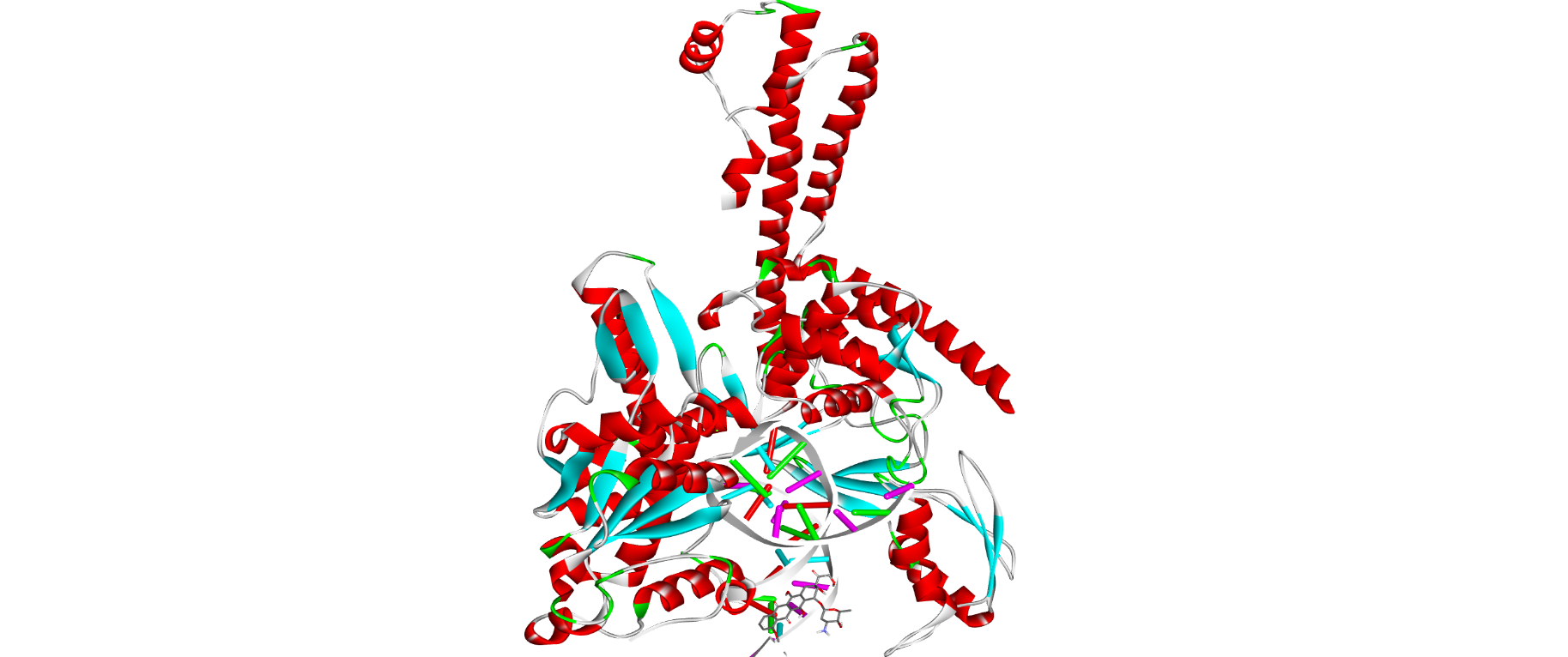 | 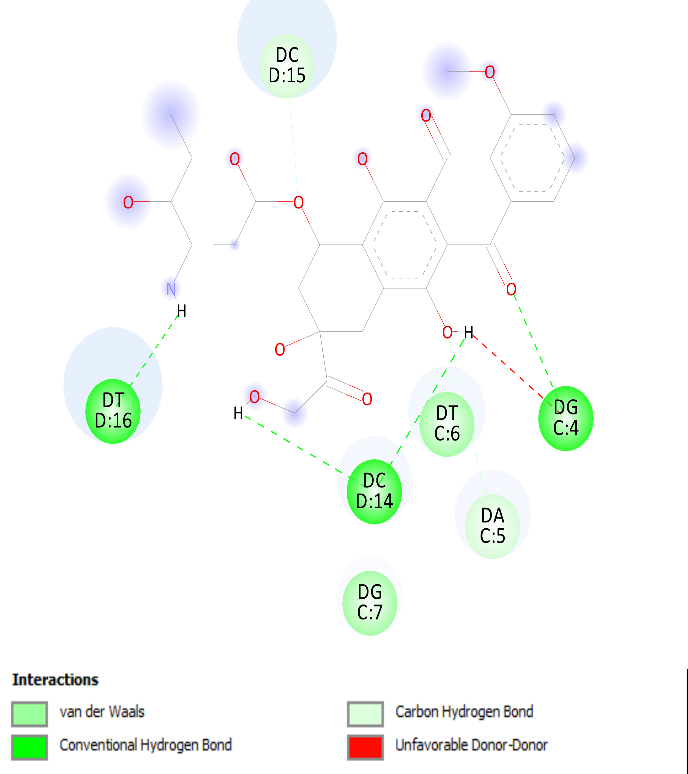 |
| **Supplementary. Fig 9.** 3D view of docking conformations of all synthesized compounds and Doxorubicin with Topoisomerase II (4fm9). | | |


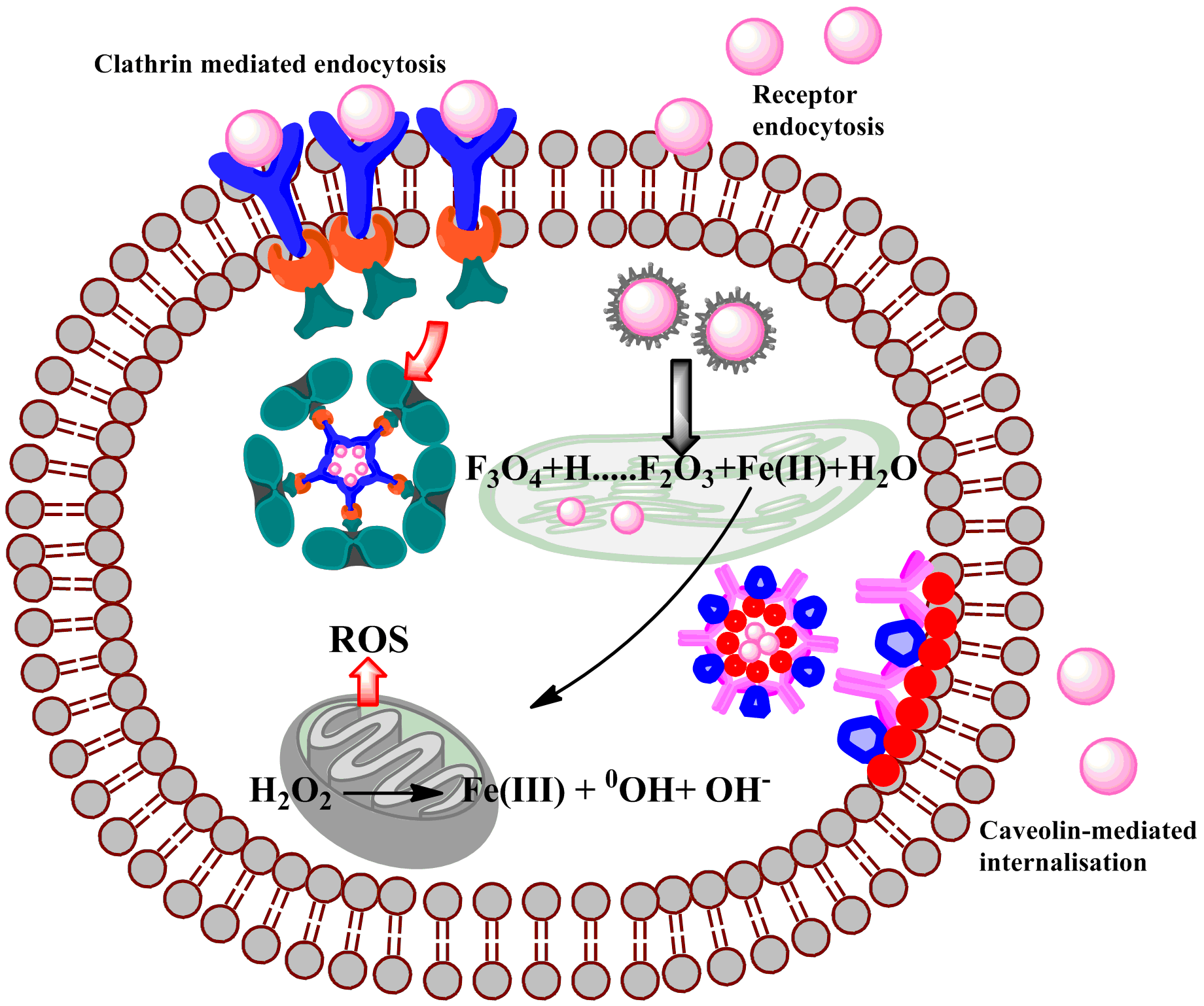


**Supplementary. Fig 10.** Schematic representation of intracellular uptake pathways and targets of INPs and Possible mechanisms of uptake of superparamagnetic iron oxide nanoparticles (SPION) include passive diffusion, clathrin-mediated endocytosis, caveolin-mediated internalisation, and other calthrin and caveolin-independent endocytosis.

1. **Corresponding author:** Ali Khaleghian Email: [khaleghian.ali@gmail.com](mailto:khaleghian.ali@gmail.com), [khaleghian@semums.ac.ir](mailto:khaleghian@semums.ac.ir),

   Address: Department of Biochemistry, Faculty of Medicine, Semnan University of Medical Sciences, 5th Km Damghan Road, Semnan, Iran- Zip Code3513138111- P.O. Box 3514533- Fax: 02333654202 [↑](#footnote-ref-1)
